# Development of a vocational rehabilitation intervention to support return-to-work and well-being following major trauma: a person-based approach

**DOI:** 10.1136/bmjopen-2024-085724

**Published:** 2024-10-04

**Authors:** Kate Radford, Jade Kettlewell, Roshan das Nair, Richard Morriss, Jain Holmes, Blerina Kellezi, Stephen Timmons, Trevor Jones, Hereward Tresidder, Isobel Andrews, Kay Bridger, Priya Patel, Rebecca Lindley, Blanca De Dios Perez, Abigail Statham, Tadeusz Jones, Karen Hoffman, Marilyn James, Denise Kendrick, ROWTATE study team

**Affiliations:** 1 Centre for Rehabilitation and Ageing Research, School of Medicine, University of Nottingham, Nottingham, UK; 2 Nottingham Biomedical Research Centre, Nottingham, UK; 3 Centre for Academic Primary Care, School of Medicine, University of Nottingham, Nottingham, UK; 4 Mental Health and Clinical Neuroscience, School of Medicine, University of Nottingham, Nottingham, UK; 5 Health Division, SINTEF, Trondheim, Norway; 6 Division of Psychology, School of Social Sciences, Nottingham Trent University, Nottingham, UK; 7 Centre for Health Innovation, Leadership and Learning, University of Nottingham Business School, Nottingham, UK; 8 Barts and the London NHS Trust, London, UK; 9 Nottingham Clinical Trials Unit, School of Medicine, University of Nottingham, Nottingham, UK

**Keywords:** rehabilitation medicine, trauma management, occupational & industrial medicine, clinical trial, implementation science, psychosocial intervention

## Abstract

**Abstract:**

**Objectives:**

Major trauma centres (MTCs) save lives but rehabilitation to support return-to-work (RTW) is lacking. This paper describes development of a vocational rehabilitation intervention (the ROWTATE intervention) to support RTW following traumatic injury.

**Design:**

Sequential and iterative person-based approach in four stages—*Stage 1:* review of evidence about the efficacy and mechanisms of RTW interventions; *Stage 2:* interviews (n=38) and focus groups (n=25) with trauma survivors and service providers in five UK MTCs to identify the issues, and challenges faced postinjury; *Stage 3*: codesign workshops (n=43) with trauma stakeholders in MTCs to conceptually test and identify intervention delivery barriers/enablers; *Stage 4:* meetings (n=7) with intervention development working group (IDWG) to: (1) generate guiding principles, (2) identify key intervention features (process, components, mechanisms) to address unmet rehabilitation needs; (3) generate a logic model and programme theory to illustrate how the intervention works; and (4) develop a training package to support delivery.

**Results:**

Trauma survivors described unmet needs relating to early advice about RTW; psychological support; pain management; hidden disabilities (eg, fatigue); estimating recovery; and community, amputee and musculoskeletal rehabilitation. Mechanisms of effective interventions identified in the review included early intervention, colocation, employer engagement, case coordination and work accommodations. Intervention features identified by IDWG members (n=13) from stages 1 and 2 were use of stepped-care approaches by occupational therapists (OTs) and clinical psychologists (CPs), OT/CP formulation for complex cases, assessment of mental health problems, individually tailored rehabilitation including vocational goal setting, cross-sector coordination/communication, employer engagement, phased RTW, education/advice for family/employers, exploration of work alternatives, ongoing review of physical and mental health needs, work stability monitoring. Conceptual testing ratified the logic model. Geography and long waiting lists were identified as potential delivery barriers.

**Conclusions:**

Real-world testing of the intervention is underway in a randomised controlled trial.

**Strengths and limitations of this study**

- Consistent with Medical Research Council guidance for complex intervention development, the person-based approach offered a systematic process for combining systematic review evidence, qualitative data reflecting wide ranging service user and provider views, with expert stakeholder input in the ROWTATE intervention design.
- Mixed methods, including systematic review and qualitative interviews ensured ROWTATE intervention components were based on the best available evidence and lived experience of >30 trauma survivors with a broad range of injuries, ages and employment types.
- The development involved codesign workshops with >70 service providers representing several healthcare professions in five geographically and socioeconomically diverse major trauma centres enabling early identification of potential implementation barriers.
- Some injury types and people from lower socioeconomic groups and ethnic minorities were under-represented in both included studies and interview data, limiting evidence for certain intervention components.
- This rich description of the intervention development, process and components will support clinical implementation if ROWTATE is found effective.

## Introduction

 In 2020/2021, more than 367 000 working aged people in England were admitted to hospital following traumatic injury or poisoning.^1^ In 2012, these injuries were estimated to cost the National Health Service (NHS) £1.53 billion each year.^2^ Major trauma is defined as an ‘*injury or combination of injuries that are life-threatening and could be life-changing because it may result in long-term disability*’.^3^ While 90% of people survive major trauma,^4^ many experience long-term physical^5^ and psychosocial^6^ difficulties that affect daily activities, including work. However, support for return-to-work (RTW) following traumatic injury is lacking.^7 8^ Reviews suggest only 41% of participants with traumatic brain injuries (TBIs),^9^ 33% of individuals with spinal cord injuries (SCIs)^10^ and 68% of orthopaedic trauma patients^11^ RTW by 1 year postinjury. Many experience anxiety, depression and post-traumatic stress disorder (PTSD) that may affect workplace productivity, sickness absence and job retention.^12 13^ Being out of work has serious financial implications for the individual, health services and society^14^ and negatively impacts physical and mental health.^12^ Supporting trauma patients to RTW is a recognised role for healthcare professionals,^15^ trauma services^16 17^ and employment is a success indicator in the NHS outcomes framework.^18^ Clinical guidelines advocate the need for vocational rehabilitation (VR), defined as a process whereby those disadvantaged by illness or disability can be enabled to access, maintain or return to employment, education or other useful occupation.^16^ However, such services are limited within the NHS and provision is patchy across the UK.^8^

While there is evidence that VR is effective for supporting RTW following TBI^19^ and for people with mental health problems^20^ or back pain,^21^ evidence to support the effectiveness of VR in general trauma populations is lacking. For people with brain injury,^19^ mental health problems^20^ and back pain^21^ effective RTW interventions have provided patients with coping skills, training and emotional support,^19^ involved both the patient and employer^20^ and taken a multidisciplinary approach including occupational therapy in addition to physical therapy.^21^ However, it remains unclear which VR intervention components and mechanisms are important to meet the needs of people following serious injury.

Medical Research Council (MRC) guidance for the development and evaluation of complex health interventions^22 23^ suggests the need for iterative, cyclical intervention development, drawing on research evidence, theory, data and stakeholder experiences with implementation issues identified at the outset, and tested during the development and evaluation stages. Hence, interventions should be ready to roll out following evaluation, thus reducing research waste.^24^ This guidance coupled with improved reporting guidelines^25^ has resulted in more transparent intervention development processes, and better intervention descriptions that facilitate replication and clinical implementation.

The person-based approach (PBA)^26^ is an iterative approach to intervention development. It involves mixed methods, incorporating relevant theory, evidence and feedback from service users and providers at each development stage for example, literature reviewing and qualitative approaches to understand user needs, and barriers and facilitators to implementation. These data are used to generate guiding principles and a logic model describing the intervention design objectives, key features and mechanisms (processes which explain how the intervention works). Intervention acceptability and feasibility can then be tested, with findings used to guide further improvements. Previous studies have used the PBA successfully to inform intervention design and optimise implementation.^27^,^30^ Given the complexity and biopsychosocial nature of VR interventions and the importance of context in their implementation,^31^ we considered PBA as the most appropriate method for designing our intervention. The relevance of a biopsychosocial perspective for RTW after trauma was demonstrated in the icfPROreha study. Kus *et al*^32^ assessed predictors of RTW at 78 weeks postdischarge from trauma rehabilitation in a prospective multicentre longitudinal study involving 761 patients with large joint injuries and complex fractures. They identified multiple psychosocial predictors of RTW, alongside health and disability-related factors, recognising the need for person-centred rehabilitation and a biopsychosocial approach to addressing RTW. Predictors included professional sector (working in the construction, architecture, surveying and building services engineering), ongoing legal disputes, financial concerns, personality traits, preaccident life satisfaction, attitude to life and demand for pension claim. Health and disability predictors were general health, current state of health, sensation of pain, limitations and restrictions in activities and participation.

This paper describes the development of a VR intervention for trauma patients delivered by occupational therapists (OTs) and clinical psychologists (CPs). The intervention was informed by our VR interventions for stroke^33^ and TBI,^34^ which we were seeking to adapt to meet the needs of people with a range of serious injuries following major trauma. This forms part of a research programme (the ROWTATE programme; NIHR, Ref: RP-PG-0617-20001), wherein the intervention has been tested for feasibility^35 36^ and acceptability^37^ and is currently being evaluated for effectiveness and cost-effectiveness in a randomised controlled trial (RCT). The objectives of the intervention development work were to: (1) identify the support needs, issues and challenges faced by people post-traumatic injury; (2) generate guiding principles for the intervention; (3) identify key intervention features (process, components and mechanisms) to address VR needs; (4) generate a logic model and programme theory to illustrate and explain how the intervention works; (5) devise a training package (schedule, manual and mentoring model) to support delivery. The intervention development is reported using the ‘ GUIDance for the rEporting of intervention Development (GUIDED)’^38^ checklist (see table 1).

**Table 1:** Guided checklist for ROWTATE intervention development

Item description	Page in manuscript where item is located	Other*
1. Report the context for which the intervention was developed	2, 3	
2. Report the purpose of the intervention development process	3	
3. Report the target population for the intervention development process	2	
4. Report how any published intervention development approach contributed to the development process	3, 4	
5. Report how evidence from different sources informed the intervention development process	4–16, 19	
6. Report how/if published theory informed the intervention development process	9	
7. Report any use of components from an existing intervention in the current intervention development process	4	
8. Report any guiding principles, people or factors that were prioritised when making decisions during the intervention development process	13, 40	
9. Report how stakeholders contributed to the intervention development process	4, 7,12, 13	
10. Report how the intervention changed in content and format from the start of the intervention development process	13, 14, 64	Online supplemental table 6
11. Report any changes to interventions required or likely to be required for subgroups	12–13,	
12. Report important uncertainties at the end of the intervention development process. Intervention development is frequently an iterative process	Discussion, 18	
13. Follow TIDieR guidance when describing the developed intervention	69–74	Online supplemental table 9
14. Report the intervention development process in an open access format		

* e.g. iIf item is reported elsewhere, then the location of this information can be stated here.

## Methods

**Figure 1:**
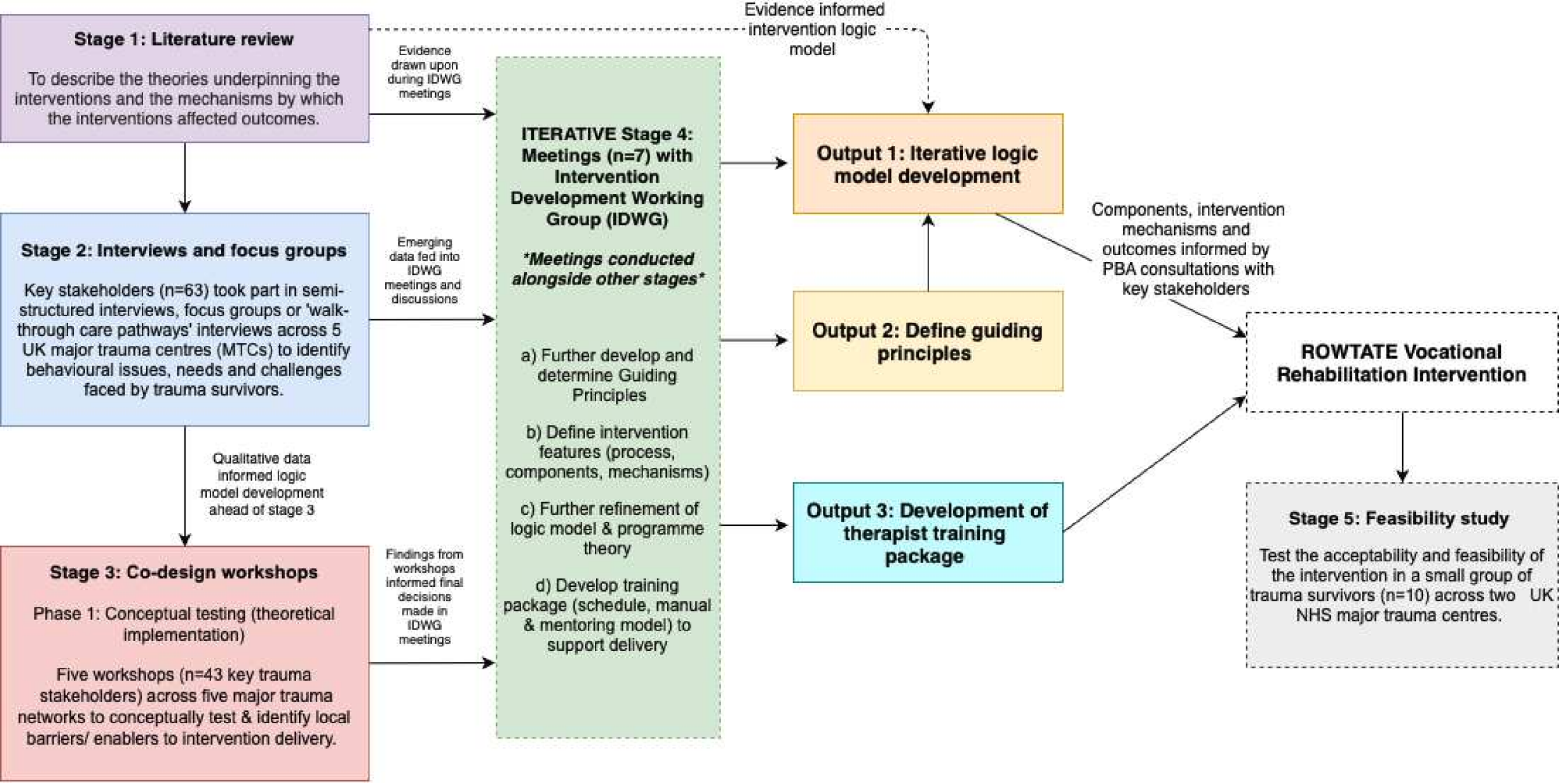
Summary of the PBA stages and data synthesis. IDWG, intervention development working group; NHS, National Health Service; PBA, person-based approach.

An iterative PBA^26^ was used comprising four stages: (1) literature review, (2) interviews, (3) codesign workshops and (4) meetings with trauma expert stakeholders in an intervention development working group (IDWG). Stages 1–3 were sequential, stage 4 was conducted in parallel with the other stages, drawing on initial findings from these stages, resulting in iterative development of the intervention and training package. A fifth stage reported elsewhere,^35^,^37^ assessed feasibility and acceptability. A summary of stages is shown in figure 1. Recruitment (for stages 2–4) started in February 2019 and lasted 12 months.

### Stage 1: literature review

We systematically searched the literature to identify RTW interventions where the primary outcome was RTW or remaining in work (job retention) or education. Where studies reported occupation-related secondary outcomes, for example work productivity, work stability, absenteeism or presenteeism rates in addition to RTW, we also reported these in order to describe the theories underpinning the interventions and the mechanisms by which the interventions affected outcomes.

We searched Medline, Embase, CINAHL, ASSIA and PsycINFO for RCTs, cohort studies and systematic reviews (to search the systematic review reference lists). Searches were conducted in June 2020. Search terms are presented in online supplemental table 2. Two independent reviewers (from a team of eight) screened titles and abstracts, assessed full texts for eligibility, extracted data and assessed risk of bias using the Cochrane RoB2 tool for RCTs^39^ and the Cochrane ROBINS-I tool for cohort studies.^40^ Disagreements at any stage were referred to a third reviewer (BK, KR). Diagrams to report the risk of bias were generated using the Robvis tool.^41^ We extracted data on study population, context (country and setting, inpatient/outpatient, staffing etc), treatment components, theory underpinning the interventions and mechanisms (processes which explain how the intervention works), so that our core intervention components were evidence-based where possible. Included studies were synthesised narratively by describing study characteristics, mechanisms, outcomes and intervention theories. Mechanisms were identified when authors attributed importance to intervention components. These were extracted verbatim and mapped to a predefined list identified from previous literature as ‘important in influencing work outcomes’.^1921 31 42^,^49^ Where authors described new previously undefined mechanisms, these were added to the list and marked as new. Interventions were mapped to one of five VR models identified in reviews by Cullen *et al*^47^ and Tyerman.^50^ These were *health focused interventions* (facilitates delivery of health services to support trauma patients’ RTW), *case and service coordination interventions* (coordination of delivery, supporting access to services and liaison with rehabilitation, vocational and community stakeholders), *work-modification interventions* (alterations to organisation of work or modifications to work), *consumer-directed interventions* (trauma patient plays a major role in running/coordination of rehabilitation) and *multidomain interventions* (interventions comprising elements from two or more of the models listed above).

### Stage 2: interviews and focus groups

Trauma survivors were recruited across five UK major trauma centres (MTCs) to represent regional variations in trauma and rehabilitation services. A sampling frame representing different types of injury was used to ensure a diverse sample of participants. Multiple recruitment strategies were used to identify a diverse sample, representative of the trauma population, including (1) existing networks of trauma survivors (‘After Trauma’ and ‘Day One’, a Leeds-based trauma charity), (2) University of Nottingham database of trauma survivors and (3) snowball sampling.^51^ Participants were approached by telephone, email or letter from the database owner. All participants provided informed consent. Interviews and focus groups were conducted by two authors (JK, KB).

To identify the key issues, support needs and challenges faced in RTW, we conducted semistructured interviews and focus groups with trauma survivors and service providers. A topic guide, informed by the International Classification of Functioning, Disability and Health (ICF),^52^ thus taking account of contextual factors affecting RTW and by our previous research in stroke^33^ and TBI^34^ (see online supplemental files 1 and 2), explored lived experiences of trauma survivors who were working or studying prior to injury, and the views of healthcare professionals with experience of delivering rehabilitation within the trauma pathway. Participants were also shown a simplified version (to facilitate understanding) of the proposed logic model and asked to comment on the intervention content, whether any changes were required to meet the needs of trauma survivors, appropriateness of intervention outcomes and whether any important outcomes were missing. To ensure inclusion of the most appropriate outcomes in the logic model, a focus group with trauma survivors (n=6) using the Nominal Group Technique^53^ identified and ranked outcomes in order of importance. Findings are reported elsewhere^54 55^

Interviews were recorded, transcribed by a University of Nottingham approved transcription service and independently analysed by three researchers (JK, KB, PP). Data were thematically analysed using Nvivo V.12^52^ adopting a staged approach, involving both inductive independent coding and theme development driven by the data to ensure trustworthiness^56 57^ and deductive coding informed by the contextual factors (environmental and personal) of the ICF^52^ to characterise biopsychosocial and contextual influences on participants RTW. The coding framework informed by the ICF is shown in online supplemental table 3.

Codes were categorised and organised into themes and subthemes, and agreed with four authors (KB, JK, KR, BK) then summarised narratively, and a map of themes and subthemes created for discussion with patient and public involvement (PPI) and the wider research team. Where disagreements arose, analyses were discussed until consensus was reached. The transcripts were revisited to ensure the accounts were coherent and accurately reflected the dataset. This combined approach ensured that coding and theme development were data-led, allowing for any important, unexpected issues to be identified, while enabling the biopsychosocial and contextual issues and challenges that trauma survivors face in returning to work to be described to inform intervention development. The summary of themes was to inform development of the guiding principles. Findings from interviews and focus groups informed the iterative development of the logic model, participant-based resource use measurement capture and outcome measures for use in the feasibility study and future RCT.

### Stage 3: codesign workshops

Five codesign workshops with key trauma stakeholders (rehabilitation service providers, people with lived experience of trauma, carers, people with experience in vocational support or rehabilitation) were conducted in five UK NHS MTCs, which were the intended sites for the RCT. Stakeholders within each major trauma network were identified and approached by known contacts of the study team, by email invite including a participant information sheet. Researchers approached stakeholders who expressed interest. Workshops lasted up to 2 hours and were facilitated by two researchers (JK, ST). The purpose of the workshops was to conduct conceptual testing of the intervention delivery (theoretical implementation). Workshop participants were shown the revised logic model and supporting intervention process flow diagram and asked to suggest changes to and identify potential issues with the proposed intervention, and local barriers/enablers to intervention delivery. Participants were also given visually mapped rehabilitation pathways specific to each major trauma network,^8^ to identify any issues within the pathway that could impact on intervention delivery. Workshop notes were analysed using the Consolidated Framework for Implementation Research (CFIR)^58^ headings, to describe and categorise implementation barriers and facilitators specific to the individual NHS Trusts. Workshop findings were brought to subsequent IDWG meetings (Stage 4) for further discussion.

### Stage 4: intervention development working group (iterative stage)

Seven IDWG meetings were held. IDWG members were VR and trauma experts, defined by ‘knowledge of traumatic injury, rehabilitation, psychological support and/or VR gained through life experience, education or training’.^59^ They included academics with expertise in VR or trauma rehabilitation (n=4), psychology (n=2) and primary care (n=1), people with lived experience of traumatic injury (n=3), a psychiatrist (n=1) and CPs (n=2). They represented potential ‘users’ of the intervention, as either recipients or involved in its delivery. IDWG members were identified via existing contacts involved in the ROWTATE study, ROWTATE study grant coapplicants, research team members and the ROWTATE PPI group.

IDWG meetings were held in parallel with the other three stages (see figure 1). Emerging findings from each stage fed into IDWG discussions. The purpose of these meetings was the iterative development of the intervention and training package. During the seven meetings, the IDWG reviewed evidence, from the systematic review (see online supplemental table 1), findings from the interviews/focus groups (stage 2) and from the codesign workshops (stage 3). The IDWG also searched and drew on National Institute for Health and Care Excellence (NICE) recommended psychological treatments for common mental health problems following trauma, including anxiety, depression, PTSD, phobias, generalised anxiety disorders, panic disorder, social anxiety, relationship breakdown, grief and bereavement^58^,^74^ and searched clinical guidelines for occupational/VR for trauma-related conditions and summarised these as resources for the training manual. This enabled us to: (1) generate guiding principles for the intervention; (2) identify the key intervention features (process, components and mechanisms) to address unmet rehabilitation needs; (3) develop a logic model and programme theory to illustrate the intervention process and explain how it might work; and (4) design a training package (schedule, manual and mentoring model) to support intervention delivery.

### Patient and public involvement

Trauma survivors as members of the ROWTATE PPI group were involved in all stages of this research.

Author TJ was a coapplicant on the grant and assisted in identifying the research questions and designing the study. Our ROWTATE PPI group, which has 14 members, meets quarterly, advising on all aspects of the research delivery. Four members of the PPI group were coopted onto the IDWG to inform the intervention development process and two were involved in training delivery. PPI members also codeveloped our interview topic guides and assisted in the design of participant recruitment materials, coproduced a quarterly newsletter and have contributed to writing this paper.

## Results

### Stage 1: literature review

**Figure 2:**
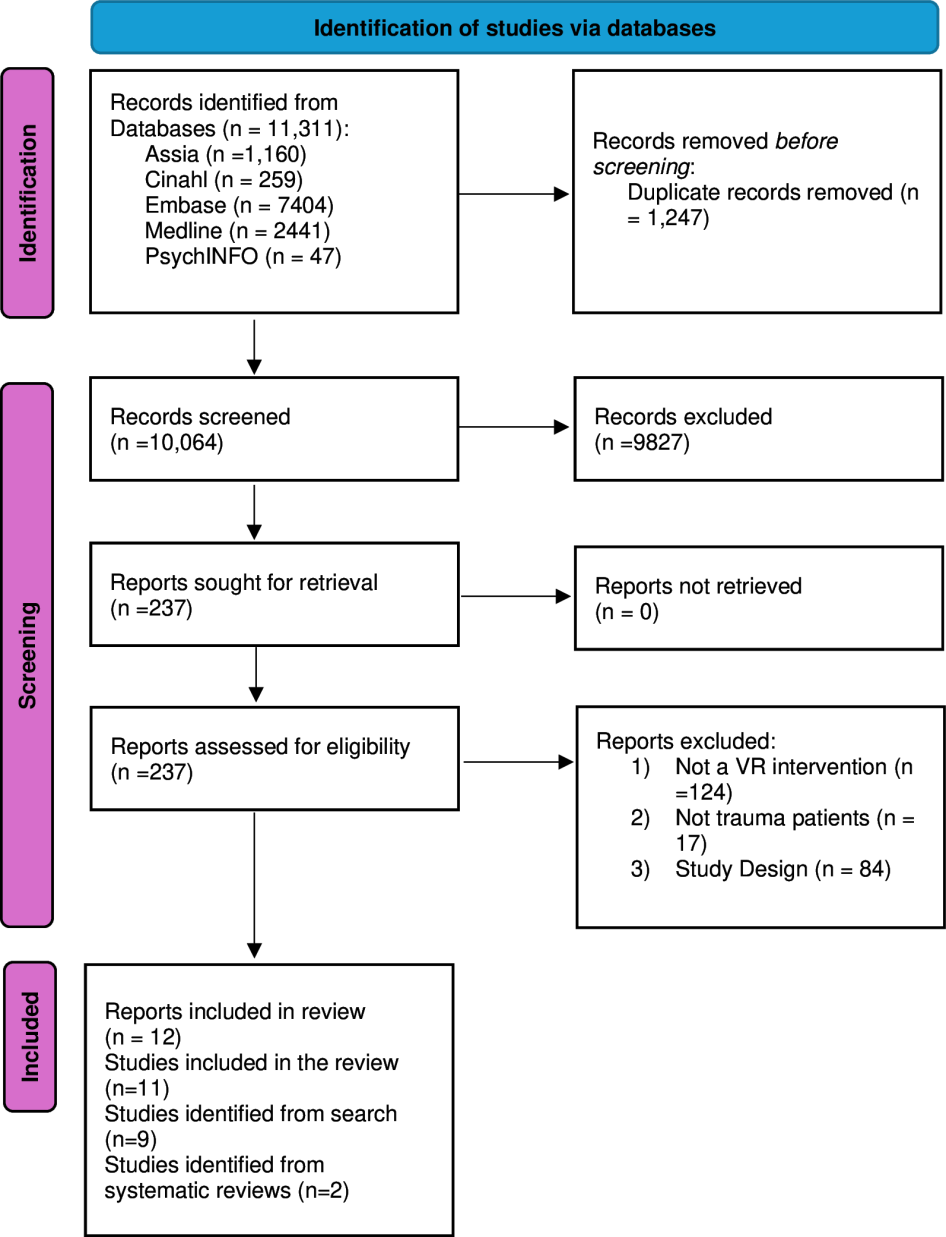
Flow chart of systematic review.

Nine RCTs and two cohort studies were included in the review. The process of study selection is shown in figure 2. Characteristics of included studies are shown in online supplemental table 1. Nine studies comprised patients with TBI),^3460^,^63 75^ one with SCI^79 80^ and one with polytrauma.^65^ Eight of the studies^3460^,^63 75 76^ had fewer than 100 participants and the maximum number of participants in any study was 201.^79 80^ Of the identified models, four were health focussed^60^,^63^; two were case coordination models^64 65^; and the rest were multidomain models.^3363 78^,^80^ There was little evidence to favour one model over another. Intervention components are shown in online supplemental table 1 and outcomes in online supplemental table 4. Three studies primarily focused on remediation of the injury (promoting recovery),^60 63 78^ whereas the others included both remediation and compensatory components (aimed at accommodating impairments in the workplace). The FRESH and NTBIS models^34 77^ were the only TBI-specific models to include both remedial and compensatory components for people with TBI.

All interventions except Man *et al*^63^ included psychological components. Four interventions offered work trials or brokered work placement^34 61 62 77^ and eight interventions provided job follow along, which involves progress and performance monitoring to ensure work stability following a return to work,.^34 61 62 64 65 77 79 80^ Eight were classified as high risk of bias^60^,^6365 75 77 79 80^; no studies were classified as having a low risk of bias. Risk of bias assessment for included studies is shown in onlinesupplemental figures 1  2.

### Theory underpinning interventions

Few studies reporting a positive effect on work outcomes^61 63 64 75 77 79 80^ named a specific underlying theory or theoretical framework, but rather offered a rationale or cited evidence of the effectiveness of specific intervention components from systematic reviews or published research. The findings suggest interventions were intended to work in different ways for different target populations. VR interventions for people with TBI^61 63 64 75 77^ cited evidence for programme-based (remediation-focussed rehabilitation in hospital/outpatient settings targeted at addressing TBI impacts using psychoeducation, behavioural and cognitive components, with intended transfer of learning to the workplace),^81^ supported employment (placing the person in work and providing mentoring and support on the job)^82^ or case coordination models of VR (coordinating efforts of different players involved in TBI rehabilitation, navigating health/workplace boundaries, signposting and advocacy) as described by Fadyl and McPherson.^83^ They included both remedial and compensatory components depending on whether their focus was primarily remedial,^60 63 78^ or both remedial and compensatory.^59 60 62 64 80^

TBI remediation-focussed interventions typically involved activities to improve cognitive function and individual or group-based psychoeducation or behaviour change interventions for example, cognitive behavioural therapy (CBT) to help the brain-injured person gain insight into their difficulties, adjust to life with a brain injury and enhance feelings of competency in dealing with workplace situations through improved relationships with others including employers, co-workers and family members.

Compensatory interventions were targeted at accommodating the person with a brain injury in the workplace, for example by negotiating workplace accommodations (changes to the job, role or responsibilities or providing equipment to enable work) with an employer. They frequently involved worksite visits and educating employers/co-workers about the impact of the injury.

Case-coordination interventions^33 34 76^ involved ensuring people could access timely support by referring to, and liaising with service providers in health, employment and other settings and/or coordinating the activities of different stakeholders. These were typically multi-disciplinary neurorehabilitation models^84^ involving numerous professionals from health and other settings (eg, employment, independent or charitable sector) and combined both remedial and compensatory components.

Two TBI-focused interventions made no impact on RTW outcomes. One compared CBT to general practitioner (GP) follow-up^78^ and another compared psychoeducation to telephone counselling for people with mild TBI.^60^ The interventions aimed to enhance patients’ feeling of competency in dealing with the consequences of mild TBI, identify and challenge unrealistic illness perceptions and improve coping style (decreasing maladaptive coping and enhancing adaptive coping). Reflecting on these outcomes, Vikane *et al*^78^ questioned whether excessive attention to symptoms and the person’s limitations in daily life, and insufficient focus on RTW could have negatively impacted on RTW. Similarly, Scheenen *et al*^60^ reflected that their intervention might have magnified patients’ perception of the injury severity, negatively impacting work.

SCIs^79 80^ and polytrauma models^65^ were predominantly compensatory and focused on adaptation to the disability, emphasising the importance of crossing service boundaries, facilitating access to VR and employment services and modifying or adjusting the work environment to accommodate the injury or disability. The RTW coordinator intervention for people with polytrauma,^65^ referenced evidence from systematic reviews supporting five factors that significantly reduce work disability duration and costs, suggesting potential mechanisms affecting work outcomes. These include, work accommodation offers, contact between the healthcare provider and workplace, workplace making early contact with worker, ergonomic worksite visits and presence of a RTW coordinator, also known as a case manager.^44 45^

### Mechanisms

In interventions that positively influenced work outcomes^61 62 64 65 75 77 79 80^ the mechanisms most frequently identified were individual tailoring and case coordination, which featured in eight interventions^61 62 64 65 75 77 79 80^; vocational goal setting and review (in seven)^61 62 64 75 77 79 80^; employer engagement (in five)^33 61 62 65 75^; identifying injury impact (in five).^61 62 64 65 77^ Colocation, which involved crossing boundaries between health and employment, was a feature of seven interventions^61 62 64 65 77 79 80^; accommodating the injury at work featured in four^61 62 65 77^; early intervention featured in four^65 77 79 80^; and work preparation also featured in three.^63 75 77^ Identified mechanisms are shown in online supplemental table 5.

### Stage 2: interviews and focus groups

A total of 63 participants consented to participate in an interview or focus group. A summary of participants for the qualitative data collection activities is shown in table 3. The key issues identified from the qualitative data were: (1) patients not routinely being asked about their emotional response to trauma, (2) rehabilitation being short term; (3) patients not being asked about their plans to return to work/education during acute treatment; (4) rapidly discharged patients having limited support; (5) community rehabilitation tending to focus on improving function and failing to address return to work; (6) health service gaps (ie, lack of vocational and psychological support) and long waiting lists slowing recovery; (7) legal rights and workplace policies being unclear to patients; (8) disability and stigma possibly affecting negotiation with employer/colleagues as they fail to understand the impact of injury (especially less visible symptoms like fatigue and anxiety); (9) healthcare not relating easily to charities, social enterprises and voluntary services.

These findings fed into IDWG discussions (stage 4) and subsequently informed the key design features of our VR intervention and training package. A summary of key issues and subsequent intervention features is shown in table 2. Detailed qualitative findings have been reported elsewhere.^8 55 85^

**Table 2:** Guiding principles

Key issues identified from existing evidence (stage 1) and qualitative data (stage 2)	Design objectives	Key design feature(s) of intervention	Mechanisms
1. Treatment focus is physical, not psychological. Patients not routinely asked about their emotional response and treatment is often provided for a short period of time.	Identifying psychological support needs over time.	OT provides early assessment and ongoing monitoring of patient’s mental health. Referral to CP if psychological support required. OT and CP liaise throughout intervention period if necessary.	Timely psychological supportUnderstanding injury impact on work Responsiveness/monitoring
2. Psychological problems often have delayed onset, which are often not picked up when a person is discharged and/or not receiving ongoing rehabilitation in routine care.			
3. Patients not asked about employment and their plans to return to work/education during acute treatment.	Identifying people employed at injury onset.	Early identification and assessment of vocational needs. Early intervention (within 12 weeks of injury), to assess impact of injury on person and job, advise about RTW and signpost to relevant services.	Early intervention
4. VR services vary a lot; some may come too late and in places may not be available at all.	Early identification of individuals requiring VR.		
5. Patients discharged quickly will miss support (eg, stabbing; less complex fracture).			
6. Failure to understand each individual reaction/adaptation to traumatic injury is unique.	Tailoring support to individual needs.	OT delivers individually tailored rehabilitation to support RTW. Explores alternatives to preinjury employment when return to pre-existing employer is not feasible or sustainable. OT considers patient needs (including ongoing medical needs) and sets patient-led goals. CP sets goals in line with patient needs and OT goals (where necessary) to ensure coordinated RTW. OT and CP communicate throughout intervention period.	Individual tailoringIdentifying work alternatives Vocational goal settingCase coordination
7. If change in capacity means an individual cannot return to previous role, there is a lack of support available to support them in identifying alternative roles.	Identifying work alternatives.		
8. Community rehabilitation often focuses on functional skills and stops short of RTW interventions.	Supporting RTW		
9. VR interventions need to understand individual’s plan for their future and assess individual goals at different points over time.	Vocational goal setting and review.		
10. Health service gaps/waiting lists slow recovery (particularly psychological support).	Ensuring timely psychological support	OT communicates in writing with all stakeholders. OT screens for mental health problems. OT case coordinates patient’s intervention. Multidisciplinary approach. Crossing boundaries between different sectors (health, social care, employment). OT signposts wider support services. OT and CP communicate throughout intervention period when necessary.	Case coordination Multidisciplinary Team (MDT) workingTimely psychological support
11. Communication and referral gaps between services mean transfer of care may be patchy.	Central point of contact for stakeholder communication.		
12. Healthcare does not relate easily to third sector.	Enabling interagency and cross boundary communication.		
13. Discharge letters to GP do not include rehab prescription.	Making GP aware of VR needs.		
14. Stress of ongoing DWP capacity assessments contributes to mental health issues.	Identifying psychological support needs over time.		
15. Legal rights/workplace policies unclear to patients.	Educating patients about the impact of injury and RTW.	OT provides ongoing education and advice to patient and family members. OT Initial assessment screens for hidden injuries and monitors impact, discussing with employer where appropriate.	Understanding injury impact on work
16. Impact of less visible injuries (mild TBI, cognitive deficits, fatigue, pain, urinary dysfunction) may interfere with RTW if not recognised.			Collective understandingEmployer engagement
17. Disability and stigma may affect negotiation with employer/colleagues.	Educating and advising employers about the impact of injury on work and how to support a RTW (including legal requirements and reasonable adjustments).	VR OT provides ongoing education, advice to employer and optimises employment environment. OT and CP mediation/ liaison with employer.	Collective understandingEmployer engagement Understanding injury impact on workAccommodating injury at work
18. Employer size/budget constraints impact motivation to accommodate injured employee needs.			
19. Lack of clarity about work tasks to be picked up during phased return.			

CPclinical psychologistDWPDepartment for Work and PensionsGPgeneral practitionerOToccupational therapistRTWreturn-to-workTBItraumatic brain injuryVRvocational rehabilitation

One focus group with trauma survivors specifically focused on identifying the outcomes of the VR intervention important to trauma survivors and used Nominal Group Technique (NGT) methods to prioritise outcomes. This is reported elsewhere.^54^ Most of the identified outcomes were measures relating to aspects of the rehabilitation process and mechanisms, rather than rehabilitation outcomes. For example, understanding the impact of injury, assessing capacity for and readiness to RTW, setting SMART (specific, measurable, achievable, relevant and time-bound) goals, facilitating reintegration to work, collaboration between key stakeholders and improving employer and employee knowledge. A sense of purpose was identified as the most important outcome and encapsulated having something to live for, a reason to get up in the morning, something purposeful to do that resulted in life satisfaction and remained relevant even if return to preinjury work was not possible. The outcomes were discussed by the IDWG and added to the logic model (See online supplemental figure 3).

### Stage 3: codesign workshops

A total of 43 trauma stakeholders and PPI representatives participated in the workshops. A summary of participants is shown in table 3. Various barriers were identified, and these were mapped to CIFR constructs (see online supplemental table 6). Few issues were specific to the intervention components but rather related to service delivery and organisational level problems such as concerns about the therapy teams’ capacity to take on additional patients as part of a research study, and whether the intervention would be funded long-term. Other implementation concerns relating to external policies, readiness for implementation and resources and culture and implementation climate are described in more detail elsewhere.^85^ Discussions around the logic model affirmed our initial thoughts; stakeholders agreed that the proposed intervention process, components and mechanisms were appropriate for the trauma population and they did not identify any issues with our proposed intervention design.

**Table 3:** Characteristics of participants in qualitative data collection activities

Participant type	Injury/profession	Interviews/focus groupsn (% total=63)*	Workshopsn (% total=43)*	Overall total (%)*
Service User	Amputation	1 (2%)		1 (1%)
	Brain injury	7 (11%)	3 (7%)	10 (9%)
	Polytrauma (including brain injury)	3 (5%)		3 (3%)
	Orthopaedic injury	11 (17%)	2 (5%)	13 (12%)
	Spinal injury	3 (5%)		3 (3%)
	Carer	2 (3%)		2 (2%)
Service provider	Case manager	3 (5%)		3 (3%)
	Clinical psychologist	7 (11%)	3 (7%)	10 (9%)
	Doctor/consultant	3 (5%)	9 (21%)	12 (11%)
	General practitioner	4 (6%)		4 (4%)
	Occupational physician	1 (2%)		1 (1%)
	Occupational psychologist	1 (2%)		1 (1%)
	Occupational therapist	7 (11%)	18 (42%)	25 (24%)
	Physiotherapist	2 (3%)	2 (5%)	4 (4%)
	Psychiatrist	1 (2%)		1 (1%)
	Speech and language therapist	1 (2%)	1 (2%)	2 (2%)
	Trauma rehabilitation coordinator/practitioner		1 (2%)	1 (1%)
	Trauma psychotherapist	1 (2%)		1 (1%)
	Psychology researcher	2 (3%)	1 (2%)	3 (3%)
	Disability employment advisor	1 (2%)	2 (5%)	3 (3%)
	Solicitor	2 (3%)		2 (2%)
	Trauma charity coordinator		1 (2%)	1 (1%)

* Percentages rounded to nearest whole number.

### Stage 4: meetings with IDWG experts (iterative stage)

The IDWG meetings were central to the PBA process, involving iterative discussions informed by the emerging findings from stages 1–3 described above. Seven meetings focused on four key outputs: (1) generating guiding principles for the intervention, (2) identifying the key intervention features, (3) developing the intervention logic model and (4) designing and developing a training package.

#### (1) Generating guiding principles for the intervention

The problems, support needs and challenges identified by trauma survivors in stage 2 interviews and focus groups were discussed by the IDWG and used to generate guiding principles for the intervention and to identify the design features needed to address them (see table 2).

#### (2) and (3) Identifying key intervention features and developing the provisional logic model

The logic model underwent iterative development following discussions at each IDWG meeting. Following stage 2 data analysis, the design features of the intervention were cross-referenced with the preliminary logic model, and emerging evidence from the literature review to ensure the intervention process and components encapsulated the desired features. Emerging themes from the interviews and from discussions of the trauma pathway maps were cross-referenced with the logic model to identify features that may or may not work based on stakeholders’ experiences and known service gaps. This revealed several anomalies, for example in the proposed timing of the early intervention. Stakeholders considered 4–6 weeks too early for some trauma patients such as those unconscious in intensive care or suffering from post-traumatic amnesia and rapid repatriation meant potential participants would likely be missed. Therefore, the inclusion criteria were modified to recruit patients up to 12 weeks postinjury and following discharge. A feature enabling participants to refer themselves back to the ROWTATE intervention within the 12-month intervention period was included to accommodate those whose recovery is delayed, for example, people awaiting amputations or having multiple surgeries. The logic model was further developed following the codesign workshops to address driving and transport issues, the involvement of GPs and other stakeholders from the employment/charitable sectors. A summary of changes to the logic model over time are shown in online supplemental table 7.

Additional mechanisms were included following IDWG discussion, these included motivating patients, and optimising hope for a successful RTW, boundary spanning (ie, crossing boundaries between health, employment, private healthcare and charitable sector service providers) and communicating with the larger multidisciplinary team. Experts felt these mechanisms were important to bringing about the necessary change to achieve outcomes.

One of the main changes involved splitting the logic model into OT-specific and CP-specific versions. Although a focus of our intervention is to encourage collaborative working between OTs and CPs and development of a joint formulation (ie, structured approach to explore in detail an individual’s situation, and understand factors affecting their mood, functioning and so on to develop a tailored intervention), it was also important to determine the components relevant to each profession separately. This helped to facilitate discussions with experts, drawing on the knowledge of different stakeholders and identify key components for the training. For example, sessions on identifying signs of psychological distress, depression and anxiety and PTSD for both CPs and OTs, a session on joint case formulation and a competency assessment adopting a team Objective Structured Clinical Examination (OSCE) design.^86^,^88^

Several meetings focused on developing a stepped care approach to treating psychological problems. The group considered that trauma patients may require different levels of support, and some might not need clinical intervention from the ROWTATE psychologist but would benefit from signposting to other local services (eg, NHS talking therapies for anxiety and depression) or may need monitoring by the OT for emerging mental health problems. This resulted in identification of different ‘levels’ of psychological intervention. At level 1, only the OT intervenes, no mental health needs are identified on screening. Level 2 participants not requiring immediate psychological intervention, but ‘at risk’ of experiencing mental health problems, are monitored by the OT for emerging mental health problems (‘watchful wait’ category) and discussed with the CP as needed. Level 3 participants exhibiting some mental health problems or scoring within the ‘borderline’ or ‘case’ threshold on the psychological assessment are assessed by the CP to determine level of intervention required. Where no psychological intervention is required, the participant is monitored by the OT for emerging mental health problems (‘Watchful wait’ category). Level 3 participants scoring within the ‘borderline or case’ threshold on psychological assessment are assessed by the CP and provided with psychological intervention from the ROWTATE CP, alongside the OT who provides the OT components of the intervention. Experts also discussed the need for referral of participants requiring psychological intervention beyond the scope of the ROWTATE intervention (eg, complex PTSD) to other mental health services (level 4). To ensure appropriate and timely involvement of the CP, the IDWG discussed the need for a screening tool comprising standardised measures of mood and PTSD, to be completed by the OT at the start of intervention delivery, with clinical cut-off scores (‘borderline’ or ‘case’) to identify participants requiring assessment by the CP. This discussion resulted in development of an intervention flow diagram (see online supplemental figure 4) describing the standardised assessment process. Decisions made and action taken by the IDWG are reported in online supplemental table 9.

#### Designing and developing a training package

Findings relating to the training of OTs and CPs from stages 1 to 3 were fed into IDWG discussions.^89^,^91^ Informed by our experiences of training OTs to deliver VR interventions in previous trials,^34 92^ and drawing on adult learning theory,^93^ the structure and content of the training were developed iteratively to reflect intervention design changes (eg, stepped care approach to psychological support), the identified learning objectives (eg, how to work collaboratively when monitoring a participant’s mental health and use formulation to address complex cases) and training needs (eg, need for OTs to be trained to identify and monitor mental health problems and administer psychological screening tools). We aimed to deliver the training in person over 2 days, with prereading and a 1-day refresher training day after 6 months. Teaching of the VR intervention components was focused on a case study, allowing us to introduce OTs and CPs to the study procedures and documentation simultaneously. OTs and CPs from each site were trained together to develop trust and shared understanding of each other’s roles. We included an overview of the trial, a taught session on PTSD, experiential group learning on how to conduct a worksite assessment and formulation and a team OSCE, comprising a standardised role play to assess competency.

CPs were encouraged to draw on NICE recommended evidence-based interventions for the treatment of anxiety,^74^ depression^70^ and PTSD.^73^ We developed a training manual, including a description of the intervention, logic model and resources to compliment the training. The IDWG agreed that OTs and CPs should be supported to deliver the intervention using VR expert-led group peer mentoring. A summary of final training components is shown in online supplemental table 8. A more detailed description of the training will be published elsewhere.

The PBA resulted in the ROWTATE intervention (see online supplemental table 10 for TIDieR description) which is a 12-month case coordinated job/education retention intervention involving OT support with vocational goal setting, provision of workplace accommodations, communication with employers, advice for the participant’s family and employer, identification of mental health problems and exploration of workplace alternatives in cases where return to the pre-existing employer is not feasible or is unsustainable. The ROWTATE OT adopts the role of case manager to overcome system, sector and process barriers using cross-sector communication. OTs and CPs work together to identify, monitor and treat mental health problems following a stepped care approach, using formulation in complex cases.

### Stage 5: evaluating acceptability and feasibility of the intervention

Stages 1–4 informed the design of the intervention, OT and CP training and mentoring, which were subsequently tested for feasibility and acceptability in a small sample of trauma patients (n=10) in two MTCs.^35^ Findings from the feasibility study will be published elsewhere.

## Discussion

We used the PBA^26 27^ and two theoretical frameworks, the ICF^52^ and CIFR,^58^ to design a complex VR intervention to address the needs of people with different types of injury, and physical, and/or psychological or employment/education-related problems, and to ensure this was grounded in a rich understanding of trauma survivor’s biopsychosocial context. To our knowledge this is the first VR intervention that has been systematically developed using a PBA, to support people with a range of traumatic injuries to return to and remain in work. Triangulation between literature review evidence, theory^52 58 94^ and in-depth qualitative research^8 55 85^ with service users and providers offered reassurance that the identified components and mechanisms were important and likely to influence outcomes relevant to this population. Combining the data and IDWG discussions enabled us to develop and refine our intervention logic model, informed by the lived experiences of trauma survivors and feedback from key stakeholders involved in rehabilitation delivery.

Although presented as a sequential stepped approach to intervention development, it was in fact iterative. Some ideas about the intervention were informed by our previous research^89 91^ and fed into this process, informing the training structure, content of the OT elements, mentoring processes and development of the topic guides. However, co-opting experts and PPI representatives to the IDWG meant our ideas were constantly challenged and the intervention changed from a more OT focused intervention to a collaborative OT and CP intervention to meet the complex and wide-ranging needs of the target population.

The literature review informed the selection of intervention components and mechanisms. No single model was identified that best suited traumatic injury VR. Those identified blended different models to address individual needs or those of diverse client groups, suggesting the need for individual tailoring and the use of both remedial and compensatory intervention components.

Our identification of mechanisms of effective interventions from previous research highlighted potential components to include in our intervention. Case coordination models mapped to mechanisms of early intervention, colocation, employer engagement, case coordination and workplace accommodations, suggesting the importance of compensatory approaches (ie, interventions targeted at the workplace/educational setting and focused on accommodating the injury and supporting the injured person in the work/education environment.^46 47 49^ These findings are consistent with mechanisms identified by Dunn *et al*^31^ in a realist review of early VR for people with acquired brain injury and SCI. They identified nine mechanisms, four of which focused on the workplace and involved engaging with employers, staying connected with the workplace, vocational goal setting and negotiating adaptations to the work role or conditions. Dunn *et al*^31^ also identified ‘fostering hope’ as a mechanism for RTW and argued that this underpins all other mechanisms. This is consistent with our NGT focus group findings in which fostering a sense of purpose was identified as the most important outcome of a RTW intervention.^54^ Dunn *et al*^31^ argue that for early VR to be effective it must be delivered within a rehabilitation team that views RTW goals as part of the initial rehabilitation following a newly acquired health condition, and be targeted towards goals that are important to the person. They suggest outcomes in the early stages of VR should include building confidence and getting the patient to recognise employment as a key goal. Dunn *et al*’s findings and our own concur with evidence from systematic reviews of workplace interventions for a range of long-term health conditions,^46 47 49^ which advocate the need for early intervention and for health services to work with employers, implement work accommodations and for coordination across service boundaries.^8^ We have therefore incorporated these mechanisms within the ROWTATE intervention in which, the OT acts as both therapist and case coordinator, intervening across the health–workplace divide and crossing service boundaries between health, social care, industry (private sector, eg, brain injury case managers and solicitors) and the workplace.

### Strengths and limitations

Our complex intervention development process had several strengths. The ROWTATE intervention components were selected based on the best available evidence, which included stakeholders’ lived experience of traumatic injury and the challenges faced in the RTW journey. Final decisions for component inclusion were made by an expert IDWG.

Our interviews and focus group participants included service users with a broad range of injuries, ages and employment types and involvement of service providers from several healthcare professions and services involved in trauma rehabilitation across five major trauma networks. We therefore elicited a wide range of views enabling a good understanding of trauma survivor’s needs, service provider perspectives and the context in which rehabilitation takes place after traumatic injury. Moreover, the inclusion of codesign workshops in five representative MTCs (eg, in terms of geographical location, socioeconomic diversity, population size, existing services), meant potential barriers to implementation (eg, poor communication between acute and community teams, beliefs about VR) were identified from the outset,^8 85^ and could be addressed prior to evaluation, thus increasing the likelihood that the intervention can be embedded in the trauma pathway. This process of involving relevant stakeholders and understanding the delivery context at the development stage is consistent with MRC guidance for complex intervention development.^24^

Another strength was our use of the ICF and CIFR to inform data collection and analysis of the qualitative data. This enabled us to consider factors likely to affect clinical implementation at the design stage, and thus maximise the potential for implementation fidelity during the clinical trial and its uptake in clinical practice (if successful), thus reducing time for translation into practice.

Consistent with guidance on developing complex interventions, the logic model underwent multiple iterations. After conceptual testing in the codesign workshops, our feasibility study undertaken in two sites^35^ offered a crucial first step for testing the feasibility of implementing the intervention and assessing its acceptability^37^ and findings from the feasibility study will be published elsewhere. Second stage usability testing is now underway in a definitive RCT^95^ with an embedded process and implementation study. This will include measurement of intervention fidelity, to determine whether the process can be followed and whether each component is necessary, so that the active ingredients (hypothesised mechanisms) can be better understood. There will also be an assessment of acceptability and barriers and facilitators to implementation from the perspectives of a wide range of stakeholders (trauma survivors, carers, OTs, CPs, GPs, employers, commissioners).

Our intervention development did have some limitations. Most evidence for VR following traumatic injury was TBI specific. Only one paper referred to polytrauma and one to SCI. The quality of the evidence was low, with none of the included studies judged to be at low risk of bias. There was very limited evidence for some intervention components, for example, psychological interventions for supporting RTW. Where psychological interventions were included, specifically related to RTW, these were mostly psychoeducational interventions for promoting insight and adjustment following TBI. However, previous longitudinal studies^96^ and our interviews with trauma survivor’s highlighted unmet need for addressing psychological problems at different time points following trauma. Therefore, we opted to include assessment of psychological problems at two key time points to ensure those who develop psychological problems later after injury, which may threaten work stability,^97^ are given timely support using evidence-based interventions for anxiety, depression and PTSD,

Some service user and service provider stakeholders were under-represented in our sample. For example, amputees, burns, polytrauma patients, carers, employers and surgeons and people from lower socioeconomic groups and ethnic minorities. The contextual relevance and acceptability of the intervention to these groups therefore needs to be better understood, and widening the stakeholders involved in the process and implementation study in the definitive RCT should help address this. Finally, qualitative data collected in stage 2 could have been mapped to ICF constructs by implementing ICF linking rules recommended by Coenen *et al* (2006)^98^ and Cieza *et al* (2019).^99^ This may have facilitated data interpretation and analysis. Future studies developing biopsychosocial interventions should consider implementing ICF linking rules to enhance communication among healthcare providers, researchers, policymakers and patients.

## Conclusions

To our knowledge this is the first VR intervention that has been systematically developed using a PBA, to support people with a range of traumatic injuries to return to and remain in work. The ROWTATE intervention has been developed in line with MRC guidance and is currently being evaluated in a definitive RCT with an embedded process and implementation study including assessment of intervention fidelity, acceptability and barriers and facilitators to implementation. Given the high incidence of traumatic injuries and their impact on work and well-being, an effective intervention would have important public health and economic implications.

## supplementary material

10.1136/bmjopen-2024-085724 online supplemental table 1

10.1136/bmjopen-2024-085724 online supplemental figure 1

10.1136/bmjopen-2024-085724 online supplemental figure 2

10.1136/bmjopen-2024-085724 online supplemental figure 3

10.1136/bmjopen-2024-085724 online supplemental figure 4

## Data Availability

Data are available upon reasonable request.
