# Supplementary material for: Development of a vocational rehabilitation intervention to support return-to-work and well-being following major trauma: a person-based approach
Source: BMJ Open. 2024 Oct 4;14(10):e085724. doi: 10.1136/bmjopen-2024-085724 (PMC11459317; doi:10.1136/bmjopen-2024-085724)
Supplement: online supplemental table 1 [file bmjopen-14-10-s001.pdf]

Supplementary files

Supplementary File 1: Example Topic guide Focus Groups

The purpose of this focus group is to gather information from key stakeholders like yourselves, to help inform and develop a return to work intervention/programme called ROWTATE. We will be discussing your experiences and opinions of current services, and any service gaps that exist. We will tell you about our proposed return to work intervention/programme called ROWTATE and ask for your feedback. This will help us develop the intervention/programme to meet the needs of people after injury, but also identify any potential barriers to delivery.
Just to remind you that this focus group will be audio recorded.
Before we begin, are there any questions you would like to ask?

1. **What services are currently available for people after trauma?**
    - → **What services does your organisation provide?**
    - → **Emotional/ Psychological/vocational/financial**
    - → **Are there any issues with access or can all trauma survivors benefit?**

2. **Does your organisation currently <u>provide</u> return to work services/support for people after trauma?**
    - → ***If so, why is this service considered to be important?***
    - → ***Trauma specific service or for a broader client group?***

3. **Thinking about the needs of people after trauma, where do you think there are service gaps?**
    - → ***How can these gaps be addressed?***

4. **Is there an unmet need for vocational support after injury?**
    - → ***Why?***
    - →

5. ***What support should be provided?***

Prompts…

Programme theory

- **What are the goals the 'programme' should hope to achieve?**
- **Which trauma related problem(s) (e.g. physical health, mental health, other) should the programme focus on?**
- **What should be done?**
- **What type(s) of programme will address the problem(s)?**
- **What's the best way of delivering these programmes?**
- **How should these be delivered?**

Thinking more specifically about the proposed ROWTATE programme…
(Present simplified ROWTATE logic model)

Programme and service delivery protocols
Programme protocol

- **What is the nature of the psychological/OT programme? (explore one or both – stakeholder dependant)**
- **What is the content of it?**

- **What is the schedule?**
- **Is it based on behaviour therapy? Another kind of therapy?**
- **Will the therapists follow standardised protocols? procedures? documents?**
- **How many sessions? How long will it last? (TiDiER detail, when, how, by whom etc)**

Service delivery protocols

- **How will a client move from screening to assessment to service delivery? (client processing/ procedures)**
- **Who is responsible for doing what? (division of labour)**
- **Where would the programme delivery take pace? (settings)**
- **Will it be group based or individual?**
- **How will the therapists communicate with each other/ clients/ other stakeholders? (Communication channels)**

Ecological Context

- **What things need to be in place to allow the programme to begin (resources)**
- **Is any type of support (resources) crucial to programme success?**
    - **E.g. Micro level- family, social support?, childcare? Transport? Materials? Macro level – community norms, cultures, Laws? Policies/ procedures? Economic factors, processes?**
    - **Are there any policies (local or national) or plans for system development that are likely to influence the programme**

Target population:

- **Which groups/ people need to be reached/ helped/ supported by this programme(s)**
    - **How should/can they be reached?**
- **What should the eligibility criteria be?**
- **When will/are the clients 'ready' to receive the programme (e.g. physical readiness, emotional readiness? Motivation to engage?)**

Program implementers

- **Who should provide the programme(s)? (therapists)**
- **Where should they come from? Which organisations should implement/ deliver the programme(s)? (organisations)**
- **What training will they require? (How will we determine, competency? (commitment, enthusiasm?)**
- **What therapist attributes might affect delivery?**

Implementing organisations

- **Does the implementing organisation have the capacity to implement this programme?**
- **How will we determine this? (Training, technology transfer, hiring experts to plan and implement)**

Associate organisations/ community partners

- **Who are the people / services the programme (our therapists) need to have working relationships with if the programme is to work?**
- **Will the 'program' require support from or collaboration with other organisations? If so, who?**
    - **Are they essential to programme delivery? (does the programme benefit from or require them?)**
    - **Do any of these organisations begin their involvement at the same time? (see our flow diagram and logic model re order of steps/ component delivery)**

Context Environment

- **Will the clients face any barriers to receiving the programmes?**
    - **If yes, what can be done to alleviate this?**
- **What environmental factors might work to support or act against implementation of the programme?**

Outcomes

- **What outcomes will be achieved by the intervention(s)/ programme?**
- **What problems will be addressed/solved? (If we do ‘this’ then what will happen as a result/ if so, then what?)**
- **Is the programme multi-level? Does it have goals at a community/ system/ society level?)**
- **What are the undesirable or unintended effects of the programme?**

Feedback Loops

- **How will we know if the program is working / on the right track? (internal/ external feedback loops)**

Supplementary File 2: Example Topic guide Interviews Trauma survivors

The purpose of this interview is to gather information from key stakeholders like yourselves, to help inform and develop a return-to-work programme called ROWTATE. We will be discussing the impact of injury, your experiences and opinions of current services, and any gaps that you feel there are in these services. We will ask you about return to work services, their purpose and why support isn't always provided/barriers to delivery. We will present our proposed return to work programme called ROWTATE and would like hear your feedback. This will help us develop the programme to meet the needs of people after injury, but also identify any potential barriers to delivery.

Before we begin, are there any questions you would like to ask?

- **What impact can injury have on a person's daily life?**
  - → ***Household tasks, caring responsibilities***
  - → ***What effect can this have on family members?***

- **What impact can injury have on a person's ability to work?**
  - → ***Paid/unpaid/self-employed***
  - → ***Time off/loss of earnings***
  - → ***How is the employer involved?***
  - → ***How do people access support?***
  - → ***What affect would this have on family members?***

- **In your experience what services are available to support people who have major injuries?**
  - → ***Psychological/vocational/financial***
  - → ***How easy are these services to access?***
  - → ***Have you accessed any of these services? If so, how?***

- **Thinking about the needs of people after major injury, do you think there are gaps in services?**
  - → ***If so what sort of gaps? Where?***
  - → ***How can these gaps be addressed?***

- **Thinking about people of working age who have major injuries, is there a need for services that support people in a return to work?**

  - → ***If yes, and we were to design a service….***

What is the problem?

- **What are the issues people who have major injuries face in returning to and remaining in work?**
- **Which of these are the most important to focus on?**
- **What needs to be done to address this problem?**
- **Who are the people who should be targeted to receive this programme?**
- **Who should deliver it?**
- **What sort of training would they need?**

- **Who should coordinate the efforts/ activities of the different people involved?**
- **Where should this support come from?**
- **What needs to be in place to make this happen?**
- **How will we know if it has been successful?**

Programme theory

- **What goals should the ‘programme’ hope to achieve?**
- **Which trauma related problem(s) (e.g. physical health, mental health, other) should the programme focus on?**
- **What should be done?**
- **What type(s) of programme will address the problem(s)?**
- **What’s the best way of delivering these programmes?**
- **How should these be delivered?**

Thinking more specifically about the proposed ROWTATE programme…
(Explain ROWTATE logic model)

- **How does this programme fit with your ideas of what is needed?/ Will it address the problem?**
  - → ***Have we included the right activities/ programmes?***
  - → ***If not, what have we missed?***
  - → ***Do the proposed outcomes look realistic?***
  - → ***Which are the most important?***

From your perspective/ experience as a service user;

- **Can you think of anything that might prevent this programme from working?**

- **Can you think of any barriers to engaging in the ROWTATE programme?**
  - → ***What would encourage you to take part?***
  - → ***What would prevent you from taking part?***

- **Do you think there may be any negative consequences**
  - → ***For the injured person?***
  - → ***For the employer?***

For the health service?

Supplementary Tables

Supplementary Table 1: Characteristics of included studies

Author	Country & Setting	Study Design	Participants	Inclusion Criteria	Program context – staffing, timing, length, mode	VR Model (Tyerman & Cullen)	Components	Mechanisms
**Traumatic Brain Injury**								
Sarajuuri et al. (2005)	Finland National Rehabilitation Centre<br><br>Post acute Inpatient setting	Cohort	39 (85% Male, aged 16-55)	• Independence in daily life and only slight physical disabilities<br>• 16 to 55 years of age<br>• Completed compulsory education.<br>• Adequate potential to achieve productivity if given special rehabilitation.	**Individualised Neuropsychological subgroup rehabilitation program (INSURE)**<br><br>Neuropsychologists, rehabilitation nurse, social worker, speech and language pathologists, OT and PT.<br>6-week face to face intervention.<br>Group and individual sessions tailored to individual needs. Each group consists of 5 to 8 members. The daily schedule runs from 8:30 to 16:00 on weekdays to simulate normal working.	Health-focused	Vocational components:<br>• Goal setting [R&C].<br><br>Psychological components:<br>• Cognitive remediation [R].<br>• Behavioural/interpersonal interventions [R].<br><br>Other components:<br>• Graded activity/exercise [R].<br>• Speech therapy [R]<br>• Therapeutic recreation [R]	• Individual tailoring.<br>• Case coordination<br>• Multi-disciplinary working<br>• Employer engagement.<br>• Responsiveness.<br>• Collective understanding<br>• Timely psychological support<br>• Vocational goal setting<br>• Identify work alternatives.<br>• Integrated treatment.
Man et al. (2013)	Hong Kong Polytechnic University<br><br>Lab based	RCT	20<br>Gender and age range not reported	• Aged between 18-55<br>• Admitted with mild to moderate TBI (mild TBI defined as GCS=13-15; PTA ≤ 1 hours; LOC ≤15 minutes, moderate TBI defined as GCS=9-12; PTA ≤ 1-24 hours; LOC ≤6 hours)<br>• Passed screening tests (Modified	**Artificial intelligent virtual reality- based vocational training system (AIVTS)**<br><br>Participants trained to use computer interface and virtual reality system then self-administered.<br><br>12 sessions (20-25 minutes per session) of clerical work pertinent to the individual including: (1) identification of office items/utilities and their proper locations; (2) handling correction fluid, files,	Multi-domain Intervention: Health-focused and Consumer Directed	Vocational components:<br>• Specific vocational skills training [R].<br><br>Other components<br>• Graded activity/exercise [R].	• Individual tailoring<br>• Work preparation

				Bathel Index, Mini-mental Status Examination and Test of Non-verbal Intelligence–Version 3)<br>• Medically stable	photocopies, printers, fax machine, desktop, cabinet, letters, and stamps, etc.; (3) work routines such as sending and receiving mail, receiving goods, managing inventory, stocktaking, etc.; and (4) office environment, safety issues and correct working posture			
Radford et al. (2013)	UK<br><br>Community	Cohort	94 (80% Male, aged 16-68)	• Aged 16+<br>• Admitted with new TBI<br>• Working paid or unpaid/in education at time of TBI	**Early TBI Specific Vocational rehabilitation**<br><br>Occupational therapist<br><br>Early target period (around 5 weeks post injury)<br><br>Lasts up to 52 weeks (individualised based on patient need)<br><br>1-2 contacts per month<br><br>Face to face, phone calls and emails	Multi-domain intervention: Health-focused, Case & service coordination and Work modification	Vocational components:<br>• Vocational assessment [R&C]<br>• Goal setting [R&C]<br>• Job analysis R&C]<br>• Vocational counselling/education [C]<br>• Work preparation [R]<br>• Vocational skills training [R]<br>• Case management/advocacy [C]<br>• Work trials [R&C]<br>• RTW planning and coordination [C]<br>• Work hardening [R&C]<br>• Job coaching [C]<br>• Job follow-along [C]<br><br>Psychological components:<br>• Cognitive remediation [R]<br>• Family counselling/education<br>• Self-responsibility and self-management type 2 [R]	• Early intervention<br>• Identify injury impact<br>• Understanding injury impact on work<br>• Individual tailoring<br>• Work preparation<br>• Co-location<br>• Accommodating injury at work<br>• Case coordination<br>• Multi-disciplinary working<br>• Employer engagement<br>• Responsiveness<br>• Accessibility<br>• Collective understanding<br>• Timely psychological support<br>• Vocational goal setting and review<br>• Identifying work alternatives<br>• Integrated treatment
Radford et al. (2018)	UK<br><br>Community	RCT	78 (85% Male, mean age 39.3, SD 13.4)	• Aged 16+<br>• Admitted with new TBI<br>• Working paid or unpaid/in education at time of TBI	**Early TBI Specific Vocational rehabilitation**<br><br>As above	Multi-domain intervention: Health-focused, Case & service coordination and Work modification	As above	As above
Twamley et al. (2014)	USA<br><br>Veteran affairs clinic or community	Pilot RCT	34<br>(94% Male, mean age 32 years)	• Operation Enduring Freedom and Operation Iraqi Freedom Veteran<br>• History of mild to moderate TBI	**Cognitive Symptom Management and Rehabilitation Therapy (CogSMART) with supported employment**	Health-focused intervention<br><br>Supported employment	Vocational components:<br>• Vocational assessment [R&C]<br>• Goal setting [R&C]<br>• Job analysis [R&C]	• Identifying injury impact<br>• Understanding injury impact on work<br>• Individual tailoring<br>• Work Preparation<br>• Case coordination

				• Impairment in at least one neuropsycho-logical domain<br>• Unemployed, but stating a goal of work	Supported employment specialists.<br><br>1 hour/week of supported employment for 1 year, plus CogSMART 1 hour/week for first 12 weeks.<br><br>Approximately 4 visits/week. Individuals or Groups<br><br>Delivered Face to face at a location of the participant's choosing (e.g., career centre, home, coffee shop, library, or Veterans Affairs clinic).		• Vocational counselling/education [C]<br>• Work preparation [R]<br>• Vocational skills/training [R]<br>• Case management/advocacy [C]<br>• Work trials [C]<br>• Job brokerage [C]<br>• Job coaching [C]<br>• Developing natural jobsite supports [C]<br>• Job follow-along [C]<br><br>Psychological components:<br>• Cognitive remediation [R]<br>• Emotional/adjustment interventions [R&C]<br>• Family counselling/education [R]<br>• Peer/group support [R]<br><br>Other components:<br>• Physical/occupational therapy [R]<br>• Speech therapy [R]<br>• Substance abuse treatment [R]<br>• Medical specialities [R]	• Multi-disciplinary working<br>• Accommodating injury at work<br>• Co-location<br>• Employer engagement<br>• Responsiveness<br>• Collective understanding<br>• Timely psychological support<br>• Vocational goal setting and review
O'Connor et al. (2016)	USA<br><br>Veteran affairs clinic or community	Pilot RCT	18 (100% Male, aged 25-69)	• Aged 18+<br>• History of mild traumatic brain injury (mTBI)<br>• Diagnosis of an MI co-occurring with mTBI.<br>• Impairment in cognitive functioning<br>• Have a "vocational problem".<br>• Potential for return to competitive employment within 6 months.	Cognitive Rehabilitation Intervention & supported employment<br><br>Cognitive Rehabilitation Specialist & VR Specialist Early target period<br><br>12-session program designed to assist in return to employment<br>1 lesson/ week.<br><br>Individual face-to-face sessions included taught (1) compensatory strategies to manage cognitive difficulties in the occupational environment	Health-focused intervention<br><br>**Supported employment**	Vocational components:<br>• Vocational assessment [R&C]<br>• Goal setting [R&C]<br>• Job analysis [R&C]<br>• Vocational counselling/education [C]<br>• Work preparation [R]<br>• Specific vocational skills training [R]<br>• Case management/advocacy [C]<br>• Work trials [C]<br>• Job brokerage [C]<br>• Job coaching [C]	• Identifying injury impact<br>• Understanding injury impact on work<br>• Individual tailoring<br>• Work Preparation<br>• Case coordination<br>• Multi-disciplinary working<br>• Accommodating injury at work<br>• Co-location<br>• Employer engagement<br>• Responsiveness<br>• Collective understanding<br>• Timely psychological support<br>• Vocational goal setting and review

				• Enrolment and participation in VR	and (2) skills to recognize and control unhelpful behaviours at work, deal with negative emotions, and foster positive relationships among coworkers and employers.<br><br>Veterans and their cognitive rehabilitation specialists met together with the Veterans' vocational rehabilitation specialists to facilitate transfer of training.		• Developing natural jobsite supports [C]<br>• Job follow-along [C]<br><br>Psychological components:<br>• Cognitive remediation [R]<br>• Emotional/adjustment interventions. [R]<br>• Family counselling/education [R]<br>• Peer/group support [R]<br>• Relationship building [R]<br>• Interpersonal skills development [R]<br>• Skills to function in life and society [R]<br><br>Other components:<br>• Physical/occupational therapy [R]<br>• Speech therapy [R]<br>• Substance abuse treatment [R]<br>• Medical specialities [R]	
Scheenen et al. (2017)	The Netherlands<br><br>Outpatient	RCT	91 (50.5% Male, aged 18-65)	• Aged between 18-65<br>• Admitted with mild TBI (defined as GCS=13-15; PTA ≤ 24 hours; LOC ≤30 minutes)<br>• Normal admission Computed tomography (CT) scan<br>• Working paid/in education at time of injury<br>• At risk for persistent posttraumatic complaints (complaints ≤3, with a least 1 in	**UPFRONT – cognitive behaviour intervention**<br><br>Healthcare psychologist<br><br>Early target period (4-10 weeks post TBI)<br>Delivered Face to face for 5 weeks: 5x1 hour small group (2-4 patients)	Health-focused intervention	Vocational components:<br>• Vocational assessment [R&C]<br>• Goal setting [R&C]<br>• Vocational counselling/education [C]<br><br>Psychological components:<br>• Emotional/adjustment intervention [R]<br>• Behavioural/interpersonal intervention [R]<br>• Self-responsibility and management type 1 and type 2 [R]<br>• Peer or group support [R]<br>• Mindfulness [R]	• Early intervention<br>• Identifying injury impact<br>• Individual tailoring<br>• Timely psychological support<br>• Vocational goal setting and review

				cognitive or emotional domain)				
Vikane et al. (2017)	Norway<br><br>Outpatients	RCT	151 (61% male, aged 16-56)	• Aged between 16-55<br>• Admitted with mild TBI (defined as GCS=13-15; PTA ≤ 24 hours; LOC ≤30 minutes), with sustained symptoms at 6-8 weeks<br>• Sick-listed or at risk to be sick list patients (at risk patients defined as reporting substantial problems at work or with moderate disability on the Extended Glasgow Outcome Scale (GOSE)	**Multidisciplinary Outpatient Programme**<br>**2 months post TBI**<br><br>Team led by specialist in rehabilitation medicine. The team included a social worker, OT, nurse, physician, GP. A few participants also met with the Norwegian Labour and Welfare Service and their employer.<br><br>Group intervention. Once a week over a consecutive 4-week period. Additional follow-ups during the first year were individually tailored to the needs of the participant.	Multi-domain intervention: Health-focused, Case & Service coordination and Work modification	Vocational components:<br>• Vocational assessment [R&C]<br>• Job analysis [R&C]<br>• Goal setting [R&C]<br>• Vocational counselling/education [C]<br>• Case management/advocacy [C]<br>• RTW planning and coordination [C].<br><br>Psychological Components:<br>• Cognitive remediation [R]<br>• Emotional/adjustment intervention [R]<br>• Peer or group support [R]<br><br>Other Components:<br>• Medical specialities [R]	• Early intervention<br>• Identifying injury impact<br>• Individual tailoring<br>• Co-location<br>• Co-ordinated effort/Case-Coordination<br>• Multi-disciplinary working<br>• Employer engagement<br>• Responsiveness<br>• Accessibility<br>• Timely psychological support<br>• Vocational goal setting & review<br>• Identify work alternatives.<br>• Integrated treatment
Trexler et al., (2016)	USA<br><br>Outpatient & Community	RCT	44 (62% male, aged 18-60)	• Aged between 18-60<br>• Native English speaker or non-native speaker with the assistance of a relative who is an English speaker or a translator<br>• TBI or diffuse encephalopathy, including metabolic, infectious, or toxic (but not because of alcohol abuse) encephalopathy, or intracranial haemorrhage.	**Resource facilitation**<br><br>Resource facilitator, Local support network leader, Clinical management team.<br><br>15-month intervention	Case and service coordination	Vocational Components:<br>• Vocational assessment [R&C]<br>• Job analysis [R&C]<br>• Goal Setting [R&C]<br>• Vocational counselling/education [R]<br>• Case management/advocacy [C]<br>• Job follow-along [C]<br><br>Psychological Components:<br>• Family counselling/education [R]<br><br>Non-specific VR:<br>• Physical/occupational therapy [R]<br>• Speech therapy [R]	• Identify Injury Impact<br>• Individual Tailoring<br>• Co-Location<br>• Co-ordinated Effort<br>• Responsiveness

				• Employed or attended school 2 years prior to injury<br>• Individual RTW or return-to-school goal<br>• Consent			• Substance abuse treatment [R]<br>• Medical specialities [R]	
**Spinal Cord Injury**								
Ottomanelli et al., 2012 & 2014	USA<br><br>Community	RCT	201 (95.5% Male, aged 13.4±2.2	• Aged 18-64<br>• SCI as a result of trauma or disease.<br>• Medical and neurological stability as determined by the principal investigator.<br>• Residence within the metropolitan area proximal to the VAMC.<br>• Access to transportation.<br>• Interest in competitive employment<br>• Willingness to complete consent form.	**Spinal Cord Injury Vocational Integration Program (SCI-VIP)**<br><br>Vocational rehabilitation counsellor.<br>Services primarily provided in the community.<br>12 months (average of 3.5 visits per participant)	Multi-Domain intervention: Health-focused, and Case & Service coordination	Vocational components:<br>• Goal setting [R&C]<br>• Case management/advocacy [C].<br>• Job brokerage [C].<br>• Job follow-along [C].<br><br>Other:<br>• Benefits counselling [C].	• Early intervention<br>• Individual tailoring<br>• Case coordination<br>• Multi-disciplinary working<br>• Responsiveness<br>• Accessibility<br>• Vocational goal setting and review<br>• Integrated treatment
**Polytrauma**								
Tan et al., (2016)	Singapore<br><br>Delivery setting not reported	RCT	152 (81.1% Female, aged 24-63)	• Workers who sustained injuries at work, which affected their RTW status. This was defined as:<br>• Admitted as inpatients in general wards or given specialist	**Return-to-work Coordinator model of care**<br><br>RTW counsellor, senior OTs and research assistants.<br><br>Early target period.<br><br>Initial assessment followed by development of a RTW plan, and active communication via	Case and service coordination	Vocational Components:<br>• Vocational assessment [R&C]<br>• Job analysis [R&C]<br>• Case management/advocacy [C]<br>• RTW planning and coordination [C].<br>• Work modifications [C]<br>• Job Coaching [C]	• Early Intervention<br>• Identify Injury Impact<br>• Understanding Injury impact on work<br>• Individual Tailoring<br>• Co-location<br>• Accommodating injury at work<br>• Case coordination<br>• Employer engagement

				outpatients appointments for further follow up, and<br>• Given more than 14 days of medical-certified leave or light duty from the date of attendance at Emergency Department.	face-to-face, telephone, or written communication to monitor progress.		• Job Follow-along [C]<br>Other Components:<br>• Physical/occupational therapy [R]<br>• Medical Specialities [R]	• Collective understanding<br>• Identify work alternatives
Key: R= Remedial; C= Compensatory; RC= Remedial and Compensatory; OT = Occupational Therapist; PT= Physiotherapist; GCS= Glasgow Coma Scale; PTA= Post Traumatic Amnesia; LOC= Loss of Consciousness. Self-responsibility and self-management type 1= Interventions focused on identifying, stage of readiness for RTW e.g. self-efficacy and decision balance, Behavioural psychological tradition; Self-responsibility and self-management type 2: Interventions focusing on enhancing coping resources and addressing non-effective ones (e.g., alcohol and substance abuse/misuse).								

Supplementary Table 2: Search terms for review

1	(Vocational OR workplace OR job) AND (rehabilitat* OR intervent* OR adjustments OR accommodat*) OR (Occupational (therap* OR medicine OR health OR health service)) OR ((Job OR work OR employment OR educat* OR volunt*) AND (maintenan* OR retention)) OR (Vocational (train* OR guidance OR educat* OR counsel*)) OR (Employment advisors OR case managers) OR (Modified (dut* OR work* OR work* environment*)) OR (Work capacity evaluation* OR work* visit) OR (Occupational (function* OR need*)) OR (Rehab* prescription)
2	(Return to (work OR employment OR education OR volunt* OR work after injur*)) OR (RTW) OR ((Phased OR gradual OR temporary) AND RTW) OR ((Job OR work* OR employment) AND (change* OR reintegration)) OR (Employ* outcome*)
3	(Traum* OR major traum*) OR ((Serious OR severe OR major OR life-threatening) AND (accident* OR injur* OR fall* OR wound*)) OR (Stab* OR burn* OR shoot* OR gun OR firearm OR bullet OR knife OR knives OR dagger OR penetrating wound OR blast injur*) OR ((Motor OR vehicle OR car OR bike OR bicycle OR cycling OR automobile) AND (accident* OR crash* OR collision*)) OR (Mva OR motor vehicle accident OR rta or road traffic accident)
4	(Traumatic Brain Injur* OR TBI) OR ((Brain OR head) AND (injur* OR ?edema OR swell* OR trauma OR h?ematoma OR h?emorrhage OR bleed* OR contusion* OR concuss* OR damage)) OR (Hypoxic brain damage OR diffuse axonal injur* or DAI*)
5	((Neck or spin*) AND (injur* OR trauma)) OR ((Para- OR quadri- OR tetra-) AND (-plegi* OR -pares*)) OR (SCI OR Spinal Cord Injur*) OR (Paralysis)
6	(Multiple OR poly) AND (traum* OR injur* OR casualt*)
7	(Injur* OR Fractur*) AND (Orthop?dic OR Upper limb OR Upper extremit* OR Lower limb* OR Lower extremit* OR Tendon* OR Joint* OR Shoulder* OR Arm* OR Elbow* OR Forearm* OR Wrist* OR Hand* OR Finger* OR Digit* OR Brachi* OR Humer* OR Radi* OR Ulna* OR Foot OR Feet OR Ankle* OR Knee* OR Leg* OR Hip* OR Fem$r* OR Tibia* OR Fibula*)
8	(1 OR 2 OR 3) AND (4 OR 5 OR 6 OR 7)
9	1. Randomized controlled trial/<br>2. Controlled clinical study/<br>3. random$.ti,ab.<br>4. randomization/<br>5. intermethod comparison/<br>6. placebo.ti,ab.<br>7. (compare or compared or comparison).ti.<br>8. ((evaluated or evaluate or evaluating or assessed or assess) and (compare or compared or comparing or comparison)).ab.<br>9. (open adj label).ti,ab.<br>10. ((double or single or doubly or singly) adj (blind or blinded or blindly)).ti,ab.<br>11. double blind procedure/<br>12. parallel group$1.ti,ab.<br>13. (crossover or cross over).ti,ab.<br>14. ((assign$ or match or matched or allocation) adj5 (alternate or group$1 or intervention$1 or patient$1 or subject$1 or participant$1)).ti,ab.<br>15. (assigned or allocated).ti,ab.<br>16. (controlled adj7 (study or design or trial)).ti,ab.<br>17. (volunteer or volunteers).ti,ab.<br>18. human experiment/<br>19. trial.ti.<br>20. or/1-19<br>21. random$ adj sampl$ adj7 (“cross section$” or questionnaire$1 or survey$ or database$1)).ti,ab. not (comparative study/ or controlled study/ or randomi?ed controlled.ti,ab. or randomly assigned.ti,ab.)<br>22. Cross-sectional study/ not (randomized controlled trial/ or controlled clinical study/ or controlled study/ or randomi?ed controlled.ti,ab. or control group$1.ti,ab.)

	23. (((case adj control$) and random$) not randomi?ed controlled).ti,ab.<br>24. (Systematic review not (trial or study)).ti.<br>25. (nonrandom$ not random$).ti,ab.<br>26. “Random field$”.ti,ab.<br>27. (random cluster adj3 sampl$).ti,ab.<br>28. (review.ab. and review.pt.) not trial.ti.<br>29. “we searched”.ab. and (review.ti. or review.pt.)<br>30. “update review”.ab.<br>31. (databases adj4 searched).ab.<br>32. (rat or rats or mouse or mice or swine or porcine or murine or sheep or lambs or pigs or piglets or rabbit or rabbits or cat or cats or dog or dogs or cattle or bovine or monkey or monkeys or trout or marmoset$1).ti. and animal experiment/<br>33. Animal experiment/ not (human experiment/ or human/)<br>34. or/21-33<br>35. 20 not 34
10	1. Meta-Analysis as Topic/<br>2. meta analy$.tw.<br>3. metaanaly$.tw.<br>4. Meta-Analysis/<br>5. (systematic adj (review$1 or overview$1)).tw.<br>6. exp Review Literature as Topic/<br>7. or/1-6<br>8. cochrane.ab.<br>9. embase.ab.<br>10. (psychlit or psyclit).ab.<br>11. (psychinfo or psycinfo).ab.<br>12. (cinahl or cinhal).ab.<br>13. science citation index.ab.<br>14. bids.ab.<br>15. cancerlit.ab.<br>16. or/8-15<br>17. reference list$.ab.<br>18. bibliograph$.ab.<br>19. hand-search$.ab.<br>20. relevant journals.ab.<br>21. manual search$.ab.<br>22. or/17-21<br>23. selection criteria.ab.<br>24. data extraction.ab.<br>25. 23 or 24<br>26. Review/

	27. 25 and 26<br>28. Comment/<br>29. Letter/<br>30. Editorial/<br>31. animal/<br>32. human/<br>33. 31 not (31 and 32)<br>34. or/28-30,33<br>35. 7 or 16 or 22 or 27<br>36. 35 not 34

Supplementary Table 3: Qualitative data coding framework linked to the ICF contextual factors

ICF Components		**Theme/code**<br>(definition)
CONTEXTUIAL FACTORS	Environmental factors	**Support**<br>(any support from healthcare professionals, carers, family members, other)
		**Environment**<br>(surrounding environment and the impact this has on RTW, providing rehab, mental and physical health)
		**Services**<br>(any services that are available to support a RTW – healthcare, social, job centre etc.)
		**Geography**<br>(location of services and support)
		**Aids**<br>(physical or technical aids in environment, workplace adjustments)
		**Adjustments**<br>(workplace adjustments, physical changes to environment,
		**Relationships**<br>(relationships between patient and therapist, employer and family)
		**Boundary spanning/co-location**<br>(links and communication between different sectors)
		**Workplace System**<br>(organisation, department, job position)
		**Healthcare system**<br>(MDT, working environment, interdisciplinary team, private practice)
		**Insurance system**<br>(personal claims etc.)
	Personal factors	**Awareness and insight**<br>(insight and self-awareness of patient on work ability, awareness of impact of injury from family and employer perspective)
		**Social function**<br>(interactions with environment, engage with work, social activities, relationships)
		**Goals**<br>(personal goals that an individual wants to achieve, either as a patient or therapist supporting patient)

		**Acceptance/Growth**<br>(acknowledging or accepting changes in functioning, moving forward)
		**Knowledge**<br>(knowledge of therapist, employer and family members about patient's work ability and how to support them, knowledge of patient to access services)
		**Impairments**<br>(impairments as a result of injury, changes in functioning)
		**Social relationships**<br>(social relationships, changes in relationship, social activities)

Supplementary Table 4. Systematic Review Primary and Secondary outcomes of included studies

Author	Control Group	Primary Work Outcome		Secondary Outcomes	
		Outcome Measure & Follow-up	Effect	Outcome Measure & Follow-up	Effect
Traumatic Brain Injury					
Sarajuuri et al. (2005)	Conventional clinical care and rehabilitation	Status of productivity; defined as working (from full-time gainful work to supported work or work trial), studying, or participating in meaningful organized voluntary work.<br>**24 months follow-up**	At 2 years follow-up 89% of the intervention group were productive compared with 55% of the controls (odds ratio_6.96; 95% confidence interval, 1.26 –38.44; P=0.017).	-	-
Man et al. (2013)	Psycho-educational Vocational Training System	Employment Outcomes; categorised as (i) return to full-time employment; (ii) return to full-time employment; (iii) return to supported employment; (iv) return to sheltered employment; and (v) being unemployed or unable to resume work.<br><br>**Follow-up at 1,3,6 months**	Within group differences (intervention and control) over three time points showed significantly more participants were in open or sheltered employment over time (intervention, P=0.04; controlP=0.0018). There was no significant difference in employment outcomes between groups over time.	WCST (computer V4; executive dysfunction)	Significant group X time interaction favouring the intervention group for:<br>Conceptual level response (P<0.001)<br>Percentage of errors (P=0.02)<br>No significant difference in:<br>Perseverative errors (P=0.56)
				Tower of London Test (Planning Problem solving)	No significant difference between groups (P=0.28)
				The Vocational Cognitive Rating Scale (Cognitive impairment in workplace)	No significant difference between groups(P=0.12)
				Self-efficacy<br>*(not collected at 6 months follow-up)*	No significant group X time interaction (P=0.33), but there was a significant difference over time for the intervention group (P=0.0014), but not for the control group (P=0.96).
				Work ability (on-site test)	No significant difference between groups (P=0.34)

Radford et al. (2013)	Usual care	Return to paid or voluntary employment of more than 1 hour a week or a return to full time education of ≥ 5 hours a week.<br><br>**Follow-up at 3,6,12 months**	3 months: 17% more intervention group participants were working than control group participants (Odds ratio= 2.00, 95% CI= 0.83, 4.83)<br>6 months: 18.2% more intervention group participants were working than control group participants (Odds ratio = 2.28, 95% CI=0.87, 5.97)<br>12 months: 15% more intervention group participants were working than control group participants (Odds ratio= 2.00, 95% CI= 0.77, 5.23)	HADS (Anxiety and depression)	No significant difference between groups (P value not reported)
				EQ-5D (Quality of Life)	No significant difference between groups (P value not reported)
				BICRO (Functional ability)	No significant difference between groups (P value not reported)
Radford et al. (2018)	Usual NHS rehabilitation	Work status; defined as competitive employment (full- or part-time paid work in an ordinary work setting, paid at the market rate.<br><br>**Follow-up 3,6,12 months**	3 Months: 51% intervention group were in competitive employment compared to 70% control group<br>6 Months: 71% intervention group in competitive employment compared to 68% control group<br>12 Months: 66% intervention group in competitive employment compared to 91% control group	HADS	*
				EQ-5D-3L	*
				Health, social care, and broader resource use	*
				WPAI v2	*
				CSI	*
				Perception of work self-efficacy (question from the WAI)	*
				NEADL (Functional ability)	*
				Participation (from CIQ)	*
				TBI recovery (GOS score) [*measured at 12 months*]	*
Twamley et al. (2014)	Enhanced Supported Employment.	Competitive employment; defined as job attainment, hours worked, and wages earned.<br><br>**14 weeks follow-up**	50% intervention group were in competitive work at 14 weeks compared to 26% control group. No significant difference between groups (P=0.15)	NSI	Intervention group improved significantly more than control group (P=0.01)
				MIST	Intervention group improved significantly more than control group (P=0.05)

				Clinician-Administered PTSD Scale (CAPS)	No difference between groups (P=0.22)
				HAM-D	No difference between groups (P=0.31)
				Quality of Life Interview-Brief Version	No difference between groups (P=0.55)
O'Connor et al. (2016)	Supportive client-centred therapy (not focus on employment or cognitive rehabilitation).	Employment defined in several ways such as working as employee, self-employment, and days of labour in exchange for pay. **12 months follow-up**	No significant difference between groups in competitive employment overall, or at 12 months or 4 months:<br><br>Overall, 50% intervention group were in competitive employment compared to 25%control group(P=0.28)<br>12 months: 50) intervention group and 12.5% of control group were in competitive employment (P=0.09).<br>4 months: no significant difference in competitive employment between groups (numbers/percentages not reported, P=0.67)	-	-
Scheenen et al. (2017)	Telephone Counselling	RTW (or study); Outcome dichotomised to 1) successful RTW (i.e., full RTW) and 2) unsuccessful RTW (i.e., partial/lower level or no RTW).<br><br>**Follow up: 3, 6, 12 Months**	No significant difference between groups at any time point (P value not reported)<br>RTW rate was 65% for intervention participants compared to 67% for control.	Functional Independence (GOSE) *[not collected at 3 months follow-up]*	Follow up 6 months: No significant difference between groups (P level not reported)<br>Follow up 12 months: Control group had significantly better functional independence than intervention group (P=0.043)
				HADS	No significant differences in mean anxiety scores over time

					for control (P=0.639) or intervention groups (P=0.357). No significant differences in mean depression scores over time for control (P=0.054) or intervention groups (P=0.452).
				The Head Injury Symptom Checklist	The control group reported significantly fewer post-traumatic complaints than the intervention group at 3 months (P=0.010) and 12 months (P=0.006), but no significant difference between groups at 6 months (P value not reported)
				Coping styles (Utrechtse Coping List)	Mean active coping scale score significantly decreased in control group (P=0.019) but not intervention group (P=0.148)
Vikane et al. (2017)	GP follow up	Days to sustainable RTW; defined as not receiving sick-leave benefits for 5 weeks<br><br>**12 months follow-up**	No significant difference between groups in RTW at 12 months after injury (P=0.173), and days sick-listed first year after injury (P=0.617)	HADS	Anxiety: No significant difference between groups (P=0.860)<br>Depression: No significant difference between groups (p=.0746)<br>Overall score: No significant difference between groups (p=0.716)
				PGIC	No significant difference between groups (P=0.285)
				Functional Independence (GOSE)	No significant difference between groups (P=0.193)

				RPQ	Total score: No significant difference between groups (P=0.096)<br>Number of symptoms:Intervention group reported fewer symptoms than control group (P=0.041)
Trexler et al. (2016)	Standard care	Vocational and academic outcome (defined as return to competitive employment, school, or volunteering) measured using the Vocational Independence Scale Revised to include levels of academic re-entry.<br>Time to RTW/community based employment<br><br>**3-,6-,9-,12-, and 15 months follow-up**	Both groups improved over time with a significant group by time interaction (P=0.027). The intervention group was on average 0.13 points higher than the control group on the Vocational Independence Scale-Revised, and at each measurement the treatment group improved 0.17 points, whereas the control group improved only 0.10 points.<br><br>Significantly more Intervention group participants RTW, school or volunteering than controls (87.5% vs 50%, P=0.024)	MPAI	No significant difference between groups (P=0.735)
				BSI-18 (GSI)	No significant difference between groups (P=0.670)
Spinal Cord Injury					
Ottomanelli et al. (2012) and (2014)	Treatment as Usual (TAU): 2 TAU groups: TAU – intervention sites (TAU-IS) and TAU-observational sites (TAU-OS)	Competitive employment; defined as "community jobs that pay at least minimum wage (paid directly by the employer to the employee) that any person can apply for, including full-time and part-time jobs".	12 months: 29.6% intervention group in employment compared to 11.8% TAU-IS (P=0.003) and 4.8% TAU-OS (P=0.002)<br>12 months: 25.9% intervention group were in competitive employment compared to 10.5% TAU-IS (P=0.008) and 2.3% TAU-OS (P=0.002)	-	-

		Ottomanelli et al. (2014): **12 months follow-up.**<br><br>Ottomanelli et al. (2014): **24 months follow-up.**	24 months: 30.8% intervention group were in competitive employment compared to 10.5% TAU-IS (P=<0.001) and 2.3% TAU-OS (P<0.002)		-
Traumatic Injury					
Tan et al. (2016)	Standard Care	Return to work status; RTW categories (whether subject returned to work with same/different employer, same/different job) and time taken to first RTW after the injury.<br><br>**3, 9 months post- work injury**	No significant difference between work status (P=0.48) or work category (P=0.27) between intervention and control groups.<br><br>Of the people who returned to work with the same employer, significantly more intervention group participants had work modifications (P = 0.04) than controls.<br><br>Participants in the intervention group took significantly less time to RTW compared to controls (P=0.029)	Quality of Life (The SF-36 v2 Standard, Singapore (English) and SF-36 v2 Standard, Singapore (Chinese) versions were used in the study)	No significant differences between groups. P values range from 0.27-0.99 across domains.
				Work Injury Notification [included incident notification rate and length of time from injury to notification]	Significantly higher notification rate in the intervention group compared to the control group (P =0 .009)

RTW: Return to Work; WCTS: Wisconsin Card Sorting Test; HADS: Hospital Anxiety and Depression Scale; EQ-5D: EuroQol 5 dimension; BICRO: The Brain Injury Community Rehabilitation Outcome scale; WPAI v2: Work Productivity and Activity Impairment; CSI: Caregiver Strain Index; WAI: Work Ability Index; CIQ: Community Integration Questionnaire; GOS: Glasgow Outcome Scale; NSI: Neurobehavioral Symptom Inventory; MSIT: Memory for Intentions Screening Test; NEADL: Nottingham Extended Activities of Daily Living; HAM-D: Hamilton Depression Rating Scale; RPQ: The Rivermead Post-Concussion Symptoms Questionnaire; PGIC: Patient's Global Impression of Change; MPAI: Mayo-Portland Adaptability Inventory; GSI: Global Severity Index; BIS-18: Brief Symptom Inventory-18; CHART: Craig Handicap Assessment and Reporting technique; QIDS-SR: Quick inventory of depressive symptomatology self-report; FIM: Functional Independence measure. ; *Feasibility study, group differences not measured; - no data on secondary outcomes.

Supplementary Table 5: Mechanisms identified in the systematic review and supporting evidence

Mechanism	Description	Evidence from Literature
**Early intervention**	Screening and early identification of trauma patients employed at time of injury ensures early advice provided to trauma patient and other involved members of the healthcare team, thus preventing decisions about relinquishing work based on injury severity symptoms/recovery. Ensures those likely to need support in returning to work are identified early in rehabilitation pathway to allow a coordinated approach to job retention, and careful planning of RTW. Ensures patients are asked about employment early on; people with less severe injuries are not missed. Promotes a coordinated approach. Prevents ill-informed advice from HCPs. Work is recognised as a health outcome after trauma by the healthcare team.<br><br>Those requiring little input may be signposted for self-management.	Ottomanelli et al., (2012); Radford et al. (2013); Ottomanelli et al., (2014); Tan et al. (2016); Scheenen et al. (2017); Vikane et al. (2017); Radford et al. (2018)
**Identifying Injury Impact**	Vocational assessment to determine the impact of injury on the person's role as a worker. May involve standardised and non-standardised assessment of physical, cognitive and psychological function and identification of personal (e.g. beliefs and attitudes) and environmental factors (workplace factors e.g. employer attitudes) that may influence work outcomes. Impact of injury on work and family identified and communicated to patient/ family/ employer.<br><br>Accurate assessment informs the intervention plan.	Radford et al. (2013); Twamley et al. (2014); O'Connor et al. (2016); Tan et al. (2016); Trexler et al. (2016); Scheenen et al. (2017); Vikane et al. (2017); Radford et al. (2018)
**Understanding injury impact on work**	Assessment of the job tasks, role and environment to evidence the intervention plan. Identifies impact of external factors or constraints to work. Raises employer awareness of injury and helps him/her think about physical and psychological aspects of the employee's role and modifications needed to support the patient's RTW. Identifies workplace factors likely to influence work outcomes (e.g. physical environment, employer attitude).	Radford et al. (2013); Twamley et al. (2014); O'Connor et al. (2016); Tan et al. (2016); Radford et al. (2018)
**Individual tailoring**	Individual tailoring of intervention components to meet trauma patient and employer needs; tailoring intervention to the context e.g. not all patients have an employer or permit employer engagement. Trauma patients may not have a job.	SaraJuuri et al. (2005); Ottomanelli et al., (2012); Man et al. (2013); Radford et al. (2013); Ottomanelli et al., (2014); Twamley et al. (2014); O'Connor et al. (2016); Tan et al. (2016); Trexler et al. (2016); Scheenen et al. (2017); Vikane et al. (2017); Radford et al. (2018)

**Work preparation**	The patient re-establishes work skills and increases work capacity by engaging in work related tasks a prior to RTW. Tasks may include real or simulated work tasks inside or outside of the workplace. For example, re-establishing work routines by simulating the working day.	Man et al. (2013); Twamley et al. (2014); O'Connor et al. (2016); Radford et al. (2013); Radford et al. (2018)
**Crossing Boundaries/ Co-location**	Crossing employment sector-health sector divide. Involves the physical transfer of information, knowledge and skills between sectors. Can occur in numerous ways; e.g. meetings that cross organisational boundaries e.g. employment sector representatives participate in clinical meetings; therapist visits workplace; multi-stakeholder communication e.g. letters/ reports; patient acts as conduit of information; providing support for the trauma patient in the workplace.	Radford et al. (2013); Twamley et al. (2014); O'Connor et al. (2016); Tan et al. (2016); Trexler et al. (2016); Radford et al. (2018)
**Accommodating injury at work**	Changes to work tasks, equipment, travel to/from work and the work environment to accommodate disability and optimise the work environment for the trauma patients' successful return. May involve supernumerary support from co-workers. Therapist negotiates workplace accommodations with employer, drawing on legislation.	Radford et al. (2013); Twamley et al. (2014); O'Connor et al. (2016); Tan et al. (2016); Radford et al. (2018)
**Case coordination**	The therapist acts as a case coordinator, involving other people/agencies in providing support. Open communication between all stakeholders ensures all those involved in supporting the RTW process remain informed and that the patient receives consistent advice. The trauma patient and other stakeholders are included in all communication and fully engaged in the RTW plan.	SaraJuuri et al. (2005); Ottomanelli et al., (2012); Radford et al. (2013); Ottomanelli et al., (2014); Twamley et al. (2014); O'Connor et al. (2016); Tan et al. (2016); Trexler et al. (2016); Vikane et al. (2017); Radford et al. (2018)
**Multi-disciplinary working**	Involves healthcare professionals working together to support people with complex care needs identified through risk stratification and case finding.	SaraJuuri et al. (2005); Ottomanelli et al., (2012); Radford et al. (2013); Ottomanelli et al., (2014); Twamley et al. (2014); O'Connor et al. (2016); Tan et al. (2016); Trexler et al. (2016); Vikane et al. (2017); Radford et al. (2018)
**Employer engagement**	Therapist liaises and negotiates with employers to facilitate a return to work. The relationship is maintained over time, creating a close working alliance. The therapist monitors and provides feedback to the employer and patient on work performance and work goals.	SaraJuuri et al. (2005); Radford et al. (2013); Twamley et al. (2014); O'Connor et al. (2016); Tan et al. (2016); Vikane et al. (2017); Radford et al. (2018)
**Responsiveness/ monitoring**	Provides longer-term monitoring, responding to changing needs (intensity, duration, components). Feedback on work performance and life goals.	SaraJuuri et al. (2005); Radford et al. (2013); Tan et al. (2016); Trexler et al. (2016); Twamley et al. (2014); O'Connor et al.

		(2016); Vikane et al. (2017); Radford et al. (2018)
**Accessibility**	Patient can easily re-access/ self-refer to the service as health needs (trauma related/co-morbidities) or employment needs (context/employer/job) change over time	Ottomanelli et al., (2012); Radford et al. (2013); Ottomanelli et al., (2014); Vikane et al. (2017); Radford et al. (2018)
**Collective Understanding**	All stakeholders have shared philosophy of intervention purpose recognising RTW as their common goal<br>Patient, family and employer understand impact of injury on person's role as a worker (limitations and abilities).<br>May involve, educating patient/family/employer about the impact of injury and educating health care professionals about work related policy, reasonable accommodations and employees' rights to ensure work is a recognised health outcome after trauma.	SaraJuuri et al. (2005); Radford et al. (2013); Tan et al. (2016); Trexler et al. (2016); Twamley et al. (2014); O'Connor et al. (2016); Radford et al. (2018)
**Timely psychological support**	Ensuring psychological support needs are identified and addressed early	SaraJuuri et al. (2005); Radford et al. (2013); Trexler et al. (2016); Twamley et al. (2014); O'Connor et al. (2016); Scheenen et al. (2017; Vikane et al. (2017); Radford et al. (2018)
**Vocational goal setting and review**	Identification, setting and review of work/ education goals with the patient (and employer/tutor where relevant). Patient motivated and hope optimised for a successful RTW	SaraJuuri et al. (2005); Ottomanelli et al., (2012); Radford et al. (2013); Ottomanelli et al., (2014); Trexler et al. (2016); Twamley et al. (2014); O'Connor et al. (2016); Scheenen et al. (2017; Vikane et al. (2017); Radford et al. (2018)
**Identifying work alternatives**	Sourcing work alternatives e.g. unpaid work, leisure where return to paid employment not possible.	SaraJuuri et al. (2005); Radford et al. (2013); Tan et al. (2016); Vikane et al. (2017); Radford et al. (2018)
**Integrated Treatment**	Vocational rehabilitation is an integral component of trauma rehabilitation rather than a bolt on or separate service.	SaraJuuri et al. (2005); Ottomanelli et al., (2012); Radford et al. (2013); Ottomanelli et al., (2014); Vikane et al. (2017); Radford et al. (2018)

Supplementary Table 6: Barriers to return to work identified during co-design workshops

	CFIR constructs	Definition of construct	Key points made during co-design workshops
Outer Setting	Patient Needs and Resources	The extent to which patient needs, as well as barriers and facilitators to meet those needs, are accurately known and prioritised by the organisation.	Some community rehabilitation teams were already providing vocational rehabilitation and/or psychological support, however waiting lists are long meaning patients' needs are not always addressed in a timely manner. Additional resources would increase therapy capacity to offer support to return to work.
	Cosmopolitanism	The degree to which an organisation is networked with other external organisations.	Major trauma centres had good links with repatriating hospitals and community teams, however stakeholders highlighted the gap in communication between acute and community care. This was highlighted as a potential barrier to implementation.
	Peer Pressure	Extent to which organisations feel peer pressure to adopt the intervention.	All participants were open to implementing the intervention in their NHS sites, however as services and processes are influenced by funding/commissioning, stakeholders felt this might be a barrier to long-term implementation.
	External Policy and Incentives	External strategies to spread interventions including policy and regulations, external mandates, recommendations and guidelines.	Stakeholders stated that policies may be a barrier to long-term implementation, but not a barrier in terms of study delivery.
Inner Setting	Structural Characteristics	How the organisation works. The social architecture, age, maturity, and size of an organisation.	Stakeholders were open to change and felt our intervention would work well within their organisation if barriers addressed.
	Networks and Communications	The nature and quality of formal and informal communications within an organisation.	Communication between healthcare professionals within the organisation and multi-disciplinary working would facilitate intervention delivery.
	Culture	Norms, values, and basic assumptions of a given organisation.	Rehabilitation stakeholders appeared open to the implementation of a vocational intervention and felt it was an important intervention.
	Implementation Climate	Absorptive capacity for change, shared receptivity of involved individuals to an intervention and the extent to which use of that intervention will be rewarded, supported and expected within their organisation.	Stakeholders agreed intervention was important for people after trauma and supported its implementation, with the hope that their organisation would encourage its delivery long-term.
	Readiness for Implementation	Tangible and immediate indicators of organisational commitment to its decision to implement an intervention.	NHS sites ready to implement the intervention for the trial.
Characteristics of individuals	Knowledge and Beliefs About the Intervention	Extent to which stakeholders know about the intervention and what do they think about it.	Stakeholders agreed that the components of the intervention were appropriate and would be feasible to deliver if service specific barriers addressed.
	Self-Efficacy	Individual belief in their own capabilities to execute courses of action to achieve implementation goals.	Stakeholders believe intervention is important and wanted to support its implementation in their NHS sites.
	Individual Stage of Change	Characterisation of the phase an individual is in, as he or she progresses toward skilled, enthusiastic, and sustained use of the intervention.	Stakeholders enthusiastic about the intervention and keen to be involved.
	Other Personal Attributes	A broad construct to include other personal traits such as tolerance of ambiguity, intellectual ability, motivation, values, competence, capacity, and learning style.	Stakeholders seemed motivated to implement the intervention in their different NHS sites.

Supplementary Table 7: Summary of logic model changes over time, following interview, focus groups and Intervention Development Working Group (IDWG) meetings.

Description And Version	Changes made as a result of qualitative data analysis and/or IDWG meetings	Reason(s) for change
Grant application logic model – original submitted.<br>**Version 1**	NA – first version	NA
Iteration 1 of logic model<br>**Version 2**	Logic model reformatted to make it simpler and in three specific sections – intervention components, mechanisms, and outcomes	Logic model format changed in preparation for interviews with key stakeholders. Creation of a simplified version for use in interviews or focus groups when questioning people about intervention components and mechanisms.
Iteration 2 of logic model<br>**Version 3**	Changes to mechanisms – added a definition (i.e. what brings about the change and how?)<br>New Mechanisms added:<br>- Education/increased knowledge:<br>• Work needs recognised by trauma teams<br>• Patient and employer understand impact of injury and make informed RTW decisions.<br>• Patient aware of available support & how to access it<br>• - Increased self-efficacy	.<br>More detail added to education section following IDWG discussion to ensure the logic model makes sense to interview/focus group participants.
Iteration 3 of logic model<br>**Version 4**<br><br>Split into two parts – OT and CP	• Logic model divided into two parts – one for Occupational Therapist (OT) and one for Clinical Psychologist (CP) to clarify the process for each therapist and distinguish components unique to each.<br>• Intervention commencement period changed from ‘up to 4 weeks post-injury’ to ‘4-12 weeks post injury’.<br>• Added goal attainment and independence to the outcomes for OT and CP logic models.<br>• CP components removed from OT logic model and added to a separate document.<br>• Added information about the OT working alongside CP to formulate a treatment plan in complex cases.<br>• CP sets goals in line with the OT	• Discussions during Programme Management Group (PMG) and interviews raised concerns about the recruitment window of only 4 weeks post-injury limiting recruitment of potentially eligible participants affected by repatriation to local hospitals or people with certain types of injury e.g. TBI where concerns exist about capacity to consent due to post traumatic amnesia (PTA).. Decision made to increase time post-injury.<br>• Following analysis of interview data and confusion around CP input and role and , their communication with the OT, the logic model was divided into two parts to facilitate interpretation and to identify mechanisms specific intervention components or combinations of components.<br>The splitting also facilitated tracking of specific changes to the OT and/or CP intervention,
Iteration 4 of OT only logic model<br>**Version 5**	• New mechanisms added:<br>○ Context and system in which OT operates (e.g. barriers and facilitators to intervention delivery)<br>○ Communication with larger Multidisciplinary team (MDT)<br>○ Co-location changed to > Boundary spanning (crossing boundaries between health, employment, independent and third sectors)<br>○ Motivating patient and optimising hope for a successful RTW.<br>○ ‘4-12 weeks post-injury’ changed to recruited ‘within 12 weeks post-injury’.	• Discussion during IDWG meeting , Co-location/boundary spanning is a key mechanism– there needs to be communication across different sectors. Health sector needs to communicate more with employment and third sectors, including between OTs and CPs.<br>• Discussion about professional boundaries and roles in the treatment and monitoring of mental health problems led to the need for more clearly defined criteria for identifying which participants need what type of support and signposting for support between OT and CP,.

	• Information added about referral to CP - OT provides ongoing mental health monitoring of patient and liaises with CP if they feel the individual requires psychological support. If CP agrees that they need support, then patient would be signposted to receive level 2, 3 or 3A psychological intervention depending on severity.<br>• Also added - VR OT works alongside clinical psychologist where necessary to develop a case formulation (i.e., if individual is receiving level 3A intervention and CP acts in consultancy role). Identifies blurred boundaries.	• Discussions during PMG and from interviews, key stakeholders felt that by having a limit of 4-12 weeks post-injury for recruitment would still mean that we miss people, specifically those that are discharged early with what seems like a less severe injury, as a result recruitment period changed to 'up to 12 weeks post-injury' to be more inclusive of target population.<br>• Information about intervention levels changed following training discussions around intervention flow diagram (see below).
Iteration 4 of CP only logic model<br>**Version 5**<br><br>Iteration 5 of OT only logic model<br>**Version 6**	• Greater sense of purpose added as an outcome.<br>• Mechanisms renamed 'crossing boundaries' instead of boundary spanning<br>• Added OT to identify key stakeholder in mechanisms<br>• CP logic model changed from 4-12 weeks post-injury to within 12 weeks of injury > recruitment period.<br>• Information added about CP monitoring mental health with OT during RTW and at 6 months.	• Changes made following focus groups focused on outcomes, intervention components, and health economics–<br>• A 'sense of purpose' was identified as the most important outcome following a nominal group focus group focused on outcomes. Therefore a measure of purpose was included as an outcome in the feasibility study.<br>• PPI asked us to change 'co-location' to 'crossing boundaries/boundary spanning', as this 'could be taken literally to imply that all of the listed entities should be physically co-located in the same office. This is clearly unrealistic'<br>• PPI also highlighted that the key stakeholder in process and component delivery be labelled –e.g by adding 'OT'.
Iteration 6 of OT only logic model<br>**Version 7**	• Information added about using psychological assessment booklet to identify whether a participant needs referral to CP. 'OT assesses mental health using booklet and refers to CP if reaches threshold, OT provides ongoing mental health monitoring and liaises with CP if they feel patient requires mental health support'.<br>• Information about watchful wait group added (i.e., level 2). States that OT will work alongside CP where necessary to develop case formation, if participant is receiving level 2 'watchful wait' intervention and CP acts in consultancy role.	• IDWG discussions about including psychological screening in the initial assessment by OT and process of signposting/referral to CP.<br>• Addition of the psychological assessment booklet and signposting to CP, need for a flow diagram indicating the assessment timing, thresholds for different levels of CP input and the process of the OT approaching and engaging the CP in the intervention to be included in the training manual.
Iteration 5 of CP only logic model<br>**Version 6**	• Information about different levels of intervention added – levels 2, 3 and 3A reflecting CP involvement depending on participants' mental health needs<br>• Signposting/referral process now detailed in the logic model. 'Participant will be referred to CP if reach threshold on psych assessment booklet'<br>• Details 'watchful wait' role and how the CP will provide support to the OT, if the OT suspects a participant needs CP intervention.	• Changes made following final IDWG meeting.<br>• Addition of the psych assessment booklet (form 17) and referral process needed to fit with flow diagram which will be added to the training manual.
Combined simplified logic model for use in the ROWTATE training manual.	• OT and CP logic models combined into a simplified version, core content remains the same, reduced words.<br>• Summary of intervention/referral levels added in key to facilitate understanding.	Simplified version of logic model created for the training manual – easier for OTs/CPs to understand.

Key: Programme Management Group = research programme management group for the Return to Work after trauma (ROWTATE) study

Supplementary Table 8: Summary of Training Package Components

Training Package Element	Training material/ session title	Content	Mode	Delivered by
ROWTATE orientation materials	Sam case study	Non-mandatory case study with questions relating to supporting RTW.	Word doc – therapists typed into and emailed back	Training lead (JH)
	ROWTATE intervention manual	Entire intervention manual to allow early familiarisation	Pdf document made available via ROWTATE website	
2-day training workshop	Introductions	Introduction to study team, mentors, OTs and CPs. Getting to know peers.	Discussion, networking	Everyone
	Return to work After Trauma (ROWTATE)	Study overview and supporting evidence	Didactic presentation with question and answer (Q&A) session	Chief Investigators (DK, KR)
	Psychological responses to traumatic accidents	Describing normal responses versus a diagnosis of Post Traumatic Stress Disorder (PTSD).	Didactic presentation with Q&A	PTSD specialist
	Trauma Specific Vocational Rehabilitation (ROWTATE Intervention)	Introduction to the four stages of the ROWTATE Intervention	Didactic presentation with Q&A	Training team (JH, JK, LP, RL, PPI)
	Overview of the CP and OT roles, research contamination	Descriptions and discussions of CP and OT roles, what is contamination in ROWTATE? and how to avoid it	Didactic presentation with open questions/discussions/reflections on current practice	
	Documenting the trial intervention	Introductions to the Case Report Forms, paper-based and cloud-based data entry and storage methods	Didactic presentation with Q&A	
	The ROWTATE Intervention process	Stage One: Early Recovery & Initial work preparation	Mix of Didactic presentations, workshop, case study, Q&A	
		Stage Two: Graded return to work		
		Stage Three: Job Retention		
		Stage Four: When return to work is not possible		
		Discharge Process		

	Competency Assessment (OSCE) with standardised role play	Professional actors performing as trauma patient. Standardised role play with case study materials and instructions for actor and therapists to follow.	Small groups/pairs. Session facilitated & filmed by training team.	
Access provided to ROWTATE website	All CRFs, documents and resources	All training materials and video recorded sessions	Password protected section of ROWTATE website with downloadable documents	Training team
Adapted element due to covid	Delivering ROWTATE via telerehabilitation	A walk through the methods and resources for online intervention delivery, video examples	Mix of didactic presentation with Q&A – live online meeting.	Training team
ROWTATE refresher training	Brief re-introductions		Discussion, networking	KR, DK, training team, CTRU
	Trial update	Providing latest news on the trial data collection, good news stories	Didactic presentation with Q&A	
	What delivering ROWTATE looks like	Review of feasibility study results – description of intervention delivery		
	Fidelity to ROWTATE and data collection	Reminder of data collection processes, examples of fidelity		

Supplementary Table 9: Focus and actions of Stage 4 IDWG meetings

Meeting number	Focus	Decisions made and actions
Month 1	• First session, deciding focus of meetings going forward.<br>• Overview of findings from qualitative interviews and considerations for training.<br>• Considerations for feasibility study.<br>• Initial discussion about content of training package.<br>• Review of preliminary logic model.	• OT and CP will share responsibility for monitoring participants' mental health over time.<br>• Provide list of local services for each site so that OTs and CPs can refer for specific or specialist interventions e.g local mental health services, limb fitting services.<br>• Split logic model into two parts (OT and CP) to clarify each intervention; re-join prior to implementation.<br>• Formulation is an important part of intervention, needs to be included in training.<br>• CP intervention needs to be split into two parts – 1) CP only and 2) CP plus OT, using formulation.<br>• Consider OSCE to assess competency of trained therapists.
Month 2	• Review new OT logic model.<br>• Discuss psychology intervention logic model in detail. Cross refer with interview data to insure guiding principles addressed.<br>• Revise training content and plan<br>• Job description criteria for OTs and CPs	• Mapping of usual care pathways should inform training package content, therapists might need training session on referral pathways for specific injury types.<br>• Training to be built around a case study, introducing the intervention and related study processes.<br>• Training plan to include more detailed description of content for each session.<br>• Clarification of case studies that will be sent out to OTs/CPs, how they will be used in the training sessions and whether any pre-training tasks will need to be completed.
Month 3	• OT and CP job descriptions<br>• Two-day training plan and delivery format<br>• Resources for therapists and manual<br>• Case studies for OTs and CPs<br>• Therapist questionnaires for the feasibility study	• Incorporate depression/mental health element into case study that will be used during the training.<br><br>The intervention should address the following issues (see also Guiding principles Table 3):<br>• Dealing with uncertainty, managing expectations and instilling hope need to be included. Helping people to come to terms with what has happened and adapt to circumstances.<br>• Recognising people have different recovery trajectories is important as these have psychological impacts. Ensure the time -frame for recovery is discussed<br>• Goal setting could be introduced into assessment & formulation.<br>• Issues re medication should be assessed and addressed e.g. including in Initial OT Assessment the question "Are you on any medication and how might this affect your ability to go back to work? E.g. safety issues re operating machinery or driving.<br>• Mentoring for OTs should address the OTs self-care, e.g., being able to refer on difficult cases to someone else.

Month 4	• Discussion of psychological component of intervention and how to engage CP in the intervention<br>• Reviewing OT and psychologist training sessions against the logic model mechanisms<br>• Therapist competency assessment<br>• Mentoring<br>• Role Checklist<br>• OT job descriptions<br>• Biopsychosocial formulation<br>• Initial Assessment checklist<br>• Self-care for OTs and CPs<br>• Review guiding principles against the issues and challenges identified in qualitative interviews	• Including peer support as part of the training package (via mentoring in peer groups)<br>• Agreed final training package content and structure.<br>• Structure training around a case study/covering the entire RTW journey and journey through the trial.<br>• Amalgamate three case studies generated to date, base around one case and add complexity to it, changing factors to discuss different treatment options.<br>• Train OTs/Cps together<br>• Send OTs/CPs a pre-training case study to ascertain understanding of VR prior to training.<br>• Introduce sessions on psychological/common mental health problems post trauma, assessing for anxiety, depression, PTSD and behavioural problems e.g. social withdrawal, lack of self-care, managing family relationships, when and when not to involve families.<br>• Invite local PTSD expert to lead session on recognising PTSD symptoms.<br>• Discuss risk issues e.g. suicide, self-harm, neglect and include in manual.<br>• Refer to NICE guidance for psychological treatment selection and methods and refer to online resources<br>• Flag the need to refer for a proper review of their medication by a relevant healthcare professional when patients raise issues about withdrawal from medication.<br>• Introduce issues re dealing with uncertainty e.g. re the recovery timeframe and RTW timescales following repeat surgery into the case studies.<br>• Triage and referral for psychological support- need to introduce a stepped care approach and flow diagram.<br>• Need to raise and discuss '0 hours' contracts, self-employed, volunteering, and sole director of a limited company as employment options and how to deal with them in training.<br>• Index the manual.<br>• Make the manual specific to the vocational rehabilitation process and refer to condition specific resources.<br>• Introduce a session on 'navigating the workplace' covering conversations with employers, highlighting different types of employment policies, negotiating phased RTW and workplace adjustments/ job accommodations.<br>• Include sessions on how the OT and CP will work together and how to use mentoring.<br>• Mentor to advise on requests for records for insurance/ compensation reports- intervention records are research data, OTs/CPs should not routinely supply these.<br>• Provide OTs/CPs with details of local services identified during mapping.
Month 5	• CP logic model<br>• Assessment and triage of patients requiring psychological support – (tools and clinical cut-offs)<br>• Clarifying Level 3 and 4 – how will review be prompted?<br>• Clarifying how OT and CP will work together<br>• Monitoring mental health and safeguarding	• Document waiting times for CP during the process evaluation.<br>• OTs may need guidance on mental health monitoring (what does 'watchful waiting' look like?). Discuss this in training.<br>• Use face to face assessment for CP<br>• Highlight the needs for OTs/CPs to be aware of their NHS Trust policies for safeguarding, noting these may differ for 16–18-year-olds.<br>• Create and include depression, anxiety and PTSD vignettes for use in training to illustrate common problems e.g. worrying, intrusive thoughts, sleep disturbance, nightmares and panic attacks and map these to the different stepped care levels and screening tool score categories.

	• What to do with serious mood disorders and people affected by the wait for psychological services<br>• What does 'watchful waiting' look like?<br>• Mentoring	
Month 6	• Use of case vignettes in training<br>• Training schedule/structure<br>• Manual content<br>• Discussion about participants aged 16-18 years – what needs adding to the intervention manual?<br>• Competency assessment<br>• CP job description<br>• Initial assessment checklist for OTs and CPs	• Include an exploring the boundaries session for CPs and OTs.<br>• Colour code the manual<br>• Include pre-reading with clear signposting on what to read, provide the vignettes in advance, map the vignettes to the ROWTATE process.<br>• Create detailed training agenda.<br>• Purchase each OT a tablet to host the manual as an electronic resource with live links to resources and assessment tools.<br>• Finalised criteria for CP involvement and CP intervention – need to revise and finalise ROWTATE process flow diagram and confirm with TMG.
Month 7	• Finalise training schedule/structure, training materials and slides<br>• Finalise competency assessment for training.<br>• Finalise initial assessment checklist for OTs/CPs<br>• Review the training case study.<br>• Discussion re goal setting how to measure goals.<br>• Review final logic model and summary of changes, highlighting points for the training<br>• Review new/ emerging evidence from systematic reviews that might influence our model	• Provide advice for OTs on how to administer and repeat use of standardised measures.<br>• Provide a leaflet on RTW that OTs can use as a calling card.<br>• PPI to be involved in training sessions.<br>• Refer to 'Manager support for RTW following sickness absence' (https://www.cipd.co.uk/) when dealing with employer questions

Supplementary Table 10: TIDieR Description of the ROWTATE Intervention

	**Description**
**Brief Name** (Provide the name or a phrase that describes the intervention.)	ROWTATE – Return to work after trauma
**WHY** Describe any rationale, theory, or goal of the elements essential to the intervention.	**Goal:** To facilitate a successful and sustained Return to Work (RTW)<br><br>• ROWTATE is a 12-month case coordinated job/education retention intervention which sets out to facilitate a successful and sustained return to work (RTW). It involves occupational therapy (OT) support with vocational goal setting, provision of workplace accommodations, communication with employers, advice for the participant's family and employer, identification of psychological problems and exploration of workplace alternatives as required.<br>• Clinical psychologists (CPs) provide psychological therapies to participant's experiencing mental health problems, following a stepped care approach.<br>• The intervention explores alternatives to pre-injury employment in cases where return to pre-existing employer is not feasible or is unsustainable.<br><br>**Underpinning Theory**<br>Intervention is underpinned by the International Classification of Function (ICF)[1], a biopsychosocial framework that considers the overall context of an individual. Importantly it takes into account the interactions between environmental (e.g., the workplace) and injury related variables, recognising that work disability is created or removed as a result of the interaction between biological, psychological, and social factors(2,3). As such, the intervention focuses on modifying work tasks and removing environmental barriers in additional to restorative approaches to promote functional recovery and psychological adjustment. We also draw on the 'Work Disability Arena' or Sherbrooke model[2] which considers the different systems (personal, workplace, healthcare and compensation system) that surround the worker, and influence return-to-work. Hence. ROWTATE adopts a case-coordinated approach to cross discipline, cross system and cross sector communication and the ROWTATE OT takes on the role of case manager.<br><br>References<br>1. *International classification of functioning, disability, and health : ICF. (2001). Geneva :World Health Organization*<br>2. *Loisel P, Durand P, Abenhaim L, Gosselin L, Simard R, Turcotte J, Esdaile JM. Management of occupational back pain: the Sherbrooke model. Results of a pilot and feasibility study. Occup Environ Med. 1994 Sep;51(9):597-602. doi: 10.1136/oem.51.9.597. PMID: 7951791; PMCID: PMC1128053.*

**WHAT**	**Materials:**<br>OTs and CPs are given ROWTATE intervention manual detailing the intervention process, content, rationale and objectives.<br><br>Additional resources are provided via the ROWTATE website (rowtate.org) via a personal log-in . These include resources targeted at employers, the participant and Ots and CPs.<br><br>• **Employers:** Chartered Institute of Personnel and Development (CIPD) line manager guide managing Return to Work (RTW), Job accommodations Network (JAN) workplace accommodation toolkit (https://askjan.org/toolkit/index.cfm), Business Disability Forum Tailored adjustments plan<br>• **Participants:** lists of services, websites, charities relevant to the major trauma centre (MTC) population<br>• **OTs and CPs** : Allied Healthcare Professionals (AHP) health and work report, physical demands characteristics chart, ROWTATE flow chart for managing participants expressing suicidal thoughts/self-harm, resources and tools to support remote rehabilitation delivery, specific resources for VR professionals, (e.g. supporting agricultural workers, site specific contacts and details of local services identified during our earlier service mapping (8), report templates and example letters: e.g. letter for a first meeting with an employer, letter heads for each site, best practice sample the OT/CP correspondence, consent to contact employer/family, discharge letter, procedure to follow when participants fail to respond or cannot be contacted, no contact example letter, OT/CP letter for ending intervention, GP update letter example, home visit risk assessment, Occupational health (OH) report example, ROWTATE assessment for work example 'chef', example RTW plans for factory cleaner/labourer/chef, RTW guidelines, session summary email example, RTW planning meeting letter example. These resources were also made available to trained OTs and CPs via the ROWTATE study website (https://www.rowtate.org.uk/).<br><br>OTs/CPs were provided with a laptop and mobile phone (if required) to conduct the remote intervention delivery sessions with study participants.<br><br>**Procedures:**<br>***Training therapists to deliver the intervention***<br>The training package comprised initial orientation materials including the intervention manual and a case study to read and answer questions. A 2-day face-to-face workshop with PowerPoint presentation was delivered by the ROWTATE training team supported by monthly mentoring from a vocational rehabilitation expert OT and CP (members of the training team).<br><br>As a result of the pandemic an additional telerehabilitation module was added in September 2020 to teach therapists how to deliver the intervention remotely and how to adapt the 'work hardening' 'workability assessment' and 'worksite assessment' components of the

	intervention for delivery by tele-rehabilitation. This was delivered remotely on MS Teams. Additional "how to" deliver rehabilitation remotely resources were provided to therapists and added to the study website. These included information about set up, and examples of work hardening plans for three types of job.<br><br>***Delivery of the intervention***<br>ROWTATE involves an OT working in a case coordinator role with a wider team of healthcare professionals, employers, family members and other agencies (e.g., solicitors, insurance and employment agencies) to:<br>• Assess the impact of the injury on the participant, family and the participant's role as a worker/student and their ability to do their job/educational course.<br>• Educate participants, employers/tutors and families about the effects of the injury and its impact on work/education and find acceptable strategies to lessen the impact.<br>• Continually monitor and assess the participant's post-injury life and work/educational goals.<br>• Prepare people for work/education by establishing structured routines with gradually increased activity levels and opportunity to practice work/study skills, e.g., structured computerised cognitive stimulation to increase concentration, daily walks to increase physical stamina.<br>• Liaise with employers/tutors, employment advisors, student services, solicitors and the healthcare team to advise about the effects of the injury and to plan and monitor a phased return to work.<br><br>At initial assessment and again at 6 months post-injury the OT screens participants for mental health problems using standardised psychological measures (GAD-2, Whooley Depression questions, Hospital Anxiety and Depression Scale, Impact of Event Scale, PHQ Panic Disorder Questionnaire). If participant scores within the 'case' or 'borderline' threshold for any of the measures, the OT approaches, involves and liaises with the CP for further assessment.<br><br>Following assessment the CP may identify no need and recommend no intervention, the CP may advise the ROWTATE OT to monitor the participant for a month then re-screen (using the psychological screen tool), the CP refers the participant to other local CP or mental health services or the ROWTATE CP delivers intervention to the participant.<br><br>Psychological interventions are delivered 1-1 and include evidence-based approaches for managing trauma-related mental health issues such as anxiety, depression and post-traumatic stress disorder (PTSD), assessment of the impact of mental health problems on work ability, teaching coping strategies, e.g., fatigue and anxiety management for use in the workplace.
**WHO PROVIDED**	**Intervention provider qualifications**<br>Health Care Professions Council (HCPC) registered occupational therapist (OT) (BSc OT) and where needed, assessment and management of psychological problems by a qualified (DClinPsy) and HCPC registered clinical psychologist (CP)<br><br>**Intervention provider background and experience**<br>• OTs with experience of working with people with serious/traumatic injuries and vocational rehabilitation (desirable)<br>• CPs with experience of delivering interventions to manage anxiety, depression and PTSD following trauma (e.g., trauma-focused CBT).<br><br>**Specific training provided**

	Pre-workshop reading materials and case study provided by email, followed by a Two-day face-to-face ROWTATE VR training session, plus two additional half-days of telerehabilitation training, provided by ROWTATE training team. This comprised an academic occupational therapist with extensive experience in delivering vocational rehabilitation, a Clinical Psychologist specialising in major trauma, supported by members of the research team with expertise in vocational rehabilitation, long-term conditions, trauma, psychology and implementation. Refresher training of x1 half day provided 6-months later where procedures were reinforced, reminders of intervention components provided and reflection of intervention delivery to date.<br><br>***Resources**<br>• Workplace accommodations from the Job Accommodations Network (JAN) https://askjan.org/<br>• Goal Attainment Scaling (GAS) https://www.kcl.ac.uk/cicelysaunders/resources/tools/gas<br>• Tailored Adjustments Plan (Business Disability Forum, 2020) Accessible via Tailored Adjustments Plans - Business Disability Forum https://businessdisabilityforum.org.uk/knowledge-hub/resources/tailored-adjustments-plans-passports-and-agreements/<br>• Allied Health Professions Fitness For Work Report (RCOT), Accessible via https://www.rcot.co.uk/practice-resources/standards-and-ethics/ahp-health-and-work-report<br>• AHP Health and Work Report: Guidance for AHP practitioners on the use and completion of the Report (Allied health Professions Federation). See; Guidance-on-completion-of-AHP-Health-and-Work-Report.pdf (ahpf.org.uk)<br>• Tailored Adjustments Plan (Business Disability Forum, 2020) Accessible via Tailored Adjustments Plans - Business Disability Forum<br>• The city of Toronto job demands analysis and job match system (Lucas, 2017), accessible via; https://silo.tips/download/the-city-of-toronto-s-job-demands-analysis-and-job-match-system<br>• Managing a return to work after long-term absence; guidance for line managers, CIPD, 2021, available at https://www.cipd.co.uk/Images/line-manager-guide-managing-return-to-work-after-long-term-absence_tcm18-97859.pdf<br>• Physical demands characteristics chart, PHYSICAL DEMAND CHARACTERISTICS OF WORK. 1993 Leonard N. Matheson,<br>• Bespoke ROWTATE resources and tools to support remote rehabilitation delivery, specific resources for VR professionals, e.g. supporting agricultural workers e.g. supporting agricultural workers http://www.agrability.org/.

**HOW**	**Mode of delivery**<br><br>The original plan was for the intervention to be delivered face to face but it was adapted for remote delivery (telerehabilitation) during the pandemic. In our feasibility study OT was delivered 90% remotely via telerehabilitation (video call or phone call) on a 1 to 1 basis. Each remote session typically lasted an hour. The CP intervention was delivered 100% remotely 1-1. In 10% of cases the CP and OT liaised directly to formulate a RTW plan. In some cases, they worked together with the employer to plan RTW. The intervention can be delivered face to face where required.<br><br>**Other**<br>Additional time spent in liaison (letters, phone and video calls) with the participant, employer, family or other healthcare providers and employment stakeholders.
**WHERE**	**Where provided**<br>intervention delivered in the community (at home or in the workplace) via telerehabilitation (via phone, video call, email, etc.) where possible. Where necessary, the OT or CP visit the participant's home or workplace should additional, and essential, in-person assessments be required.<br><br>Participant's who are still in hospital or in a rehabilitation unit when the intervention begins may be seen in person or remotely.<br><br>**Necessary infrastructure**<br>Access to laptops and local NHS approved delivery platform e.g Cisco WebEX, Accurex for OTs CPs<br>Telephone, Smartphone, laptop or tablet with phone line and or internet connection for participants and OTs, CPs
**WHEN and HOW MUCH**<br>Describe the number of times the intervention was delivered and over what period of time including the number of sessions, their schedule, and their duration, intensity or dose.	**Intervention delivery time**<br>Intervention commences within 12 weeks of injury and continues for up to 12m post randomisation. Duration and frequency tailored to individual need.<br><br>**Number of sessions and length**<br>Each intervention session will last for approximately 1 hour.<br>The length of intervention will not extend beyond 12 months.<br>Total time for intervention delivery estimated at 20 per participant.<br><br>**Frequency of sessions**<br>As determined by participant need.<br><br>In our feasibility study the 10 participants had an average of 13 sessions of OT over 12 months (median 35 weeks), sessions lasted a mean of 64 minutes with a mean total off 14.25 hours of intervention per participant. The CP intervention was delivered to 7 participants. It lasted a median of 7.9 weeks with an average of 3 sessions, lasting 50 minutes per participant and a mean total of 3.5 hours per participant.

**TAILORING**	The OT and CP interventions will be tailored in duration, content and frequency according to individual need over a 12-month period.
**MODIFICATIONS**<br>If the intervention was modified during the course of the study, describe the changes (what, why, when, and how).	Prior to starting the intervention delivery in October 2020, the intervention was adapted from face-to-face delivery to remote delivery as a result of the COVID-19 pandemic.<br><br>The intervention is designed to be individually tailored for each individual, but no modifications were made during the course of the study.
**HOW WELL**<br>Planned: If intervention adherence or fidelity was assessed, describe how and by whom, and if any strategies were used to maintain or improve fidelity, describe them.	**Planned**<br>Monthly mentoring sessions with OTs/CPs led by experienced VR OT and clinical neuropsychologist to identify implementation and fidelity issues, and discuss adherence to protocol and addressing implementation barriers and contextual and process issues related to intervention delivery. Measured by completion of intervention delivery CRFs and completion of mentoring records 0-12 months post-participant recruitment.<br><br>Fidelity assessed by checklist and quantitatively using content CRFs, qualitative data from records and interviews used to identify those factors that moderate fidelity.
Actual: If intervention adherence or fidelity was assessed, describe the extent to which the intervention was	**Actual**<br>To be added following trial completion.

delivered as planned.	
