# Supplementary material for: Development of a vocational rehabilitation intervention to support return-to-work and well-being following major trauma: a person-based approach
Source: BMJ Open. 2024 Oct 4;14(10):e085724. doi: 10.1136/bmjopen-2024-085724 (PMC11459317; doi:10.1136/bmjopen-2024-085724)
Supplement: online supplemental figure 2 [file bmjopen-14-10-s003.pdf]

**Supplementary Figure 2  Risk of Bias Assessment Cohort studies**

| Study | D1 | D2 | D3 | D4 | D5 | Overall |
|---|---|---|---|---|---|---|
| Ottomaneli et al., 2012 & 2014 | - | + | - | - | - | X |
| Man et al., 2013 | - | - | + | - | - | X |
| Twamley et al., 2014 | - | - | + | - | - | X |
| O'connor et al., 2016 | - | - | + | - | X | X |
| Tan et al., 2016 | + | - | + | - | - | X |
| Trexler et al., 2016 | + | + | + | + | - | - |
| Scheenen et al., 2017 | - | - | - | - | - | X |
| Vikane et al., 2017 | + | + | + | + | - | - |
| Radford et al., 2018 | + | + | - | + | + | - |

Risk of bias domains

Domains:
D1: Bias arising from the randomization process.
D2: Bias due to deviations from intended intervention.
D3: Bias due to missing outcome data.
D4: Bias in measurement of the outcome.
D5: Bias in selection of the reported result.

Judgement
X High
- Some concerns
+ Low
