# Supplementary figures and images for: Development of a vocational rehabilitation intervention to support return-to-work and well-being following major trauma: a person-based approach
Source: BMJ Open. 2024 Oct 4;14(10):e085724. doi: 10.1136/bmjopen-2024-085724 (PMC11459317; doi:10.1136/bmjopen-2024-085724)

**Supplementary Figure 3: ROWTATE Logic Model**

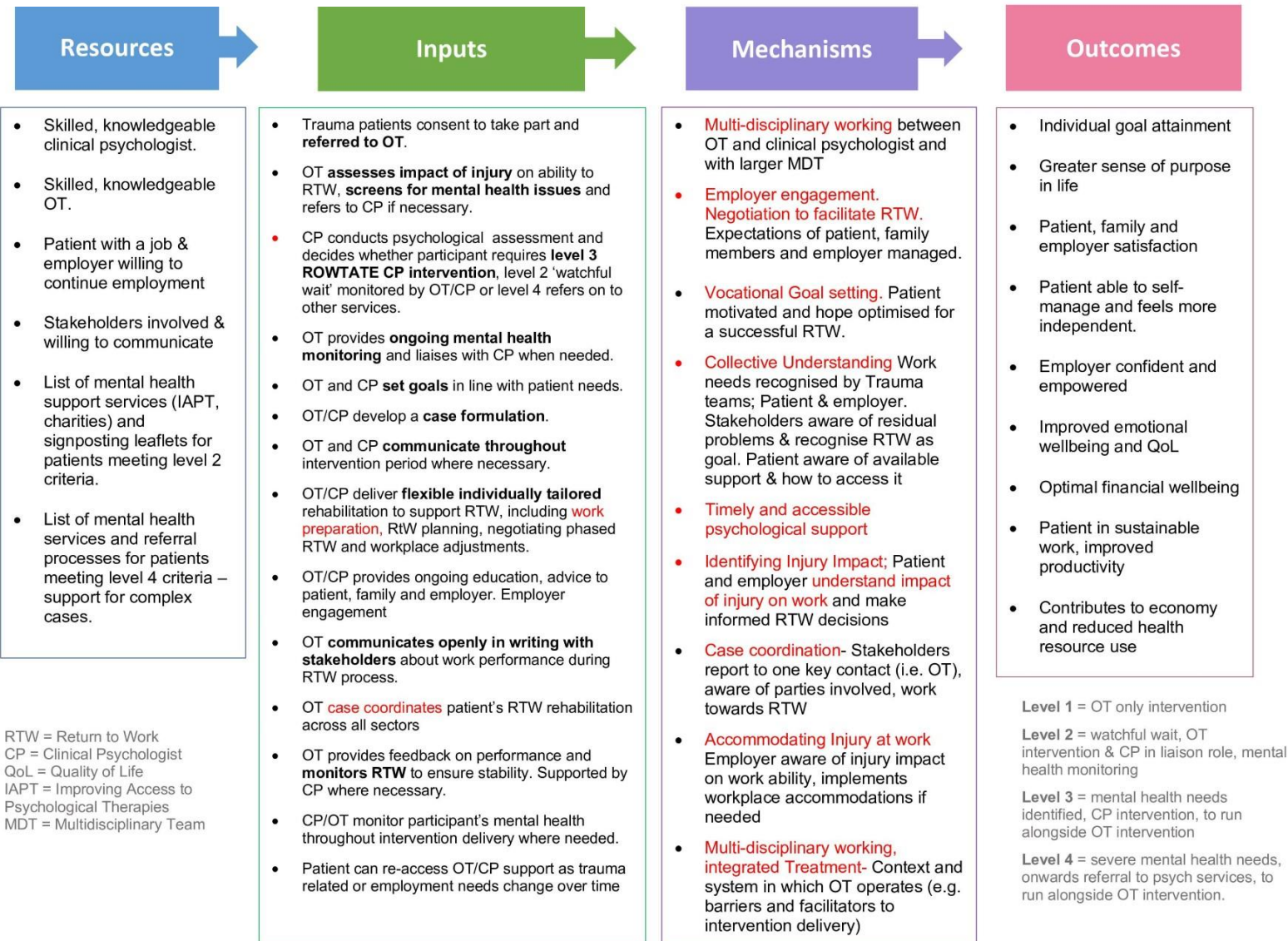

Supplement: online supplemental figure 3 [file bmjopen-14-10-s004.pdf]

**Supplementary Figure 4: Flow diagram indicating psychological screening**

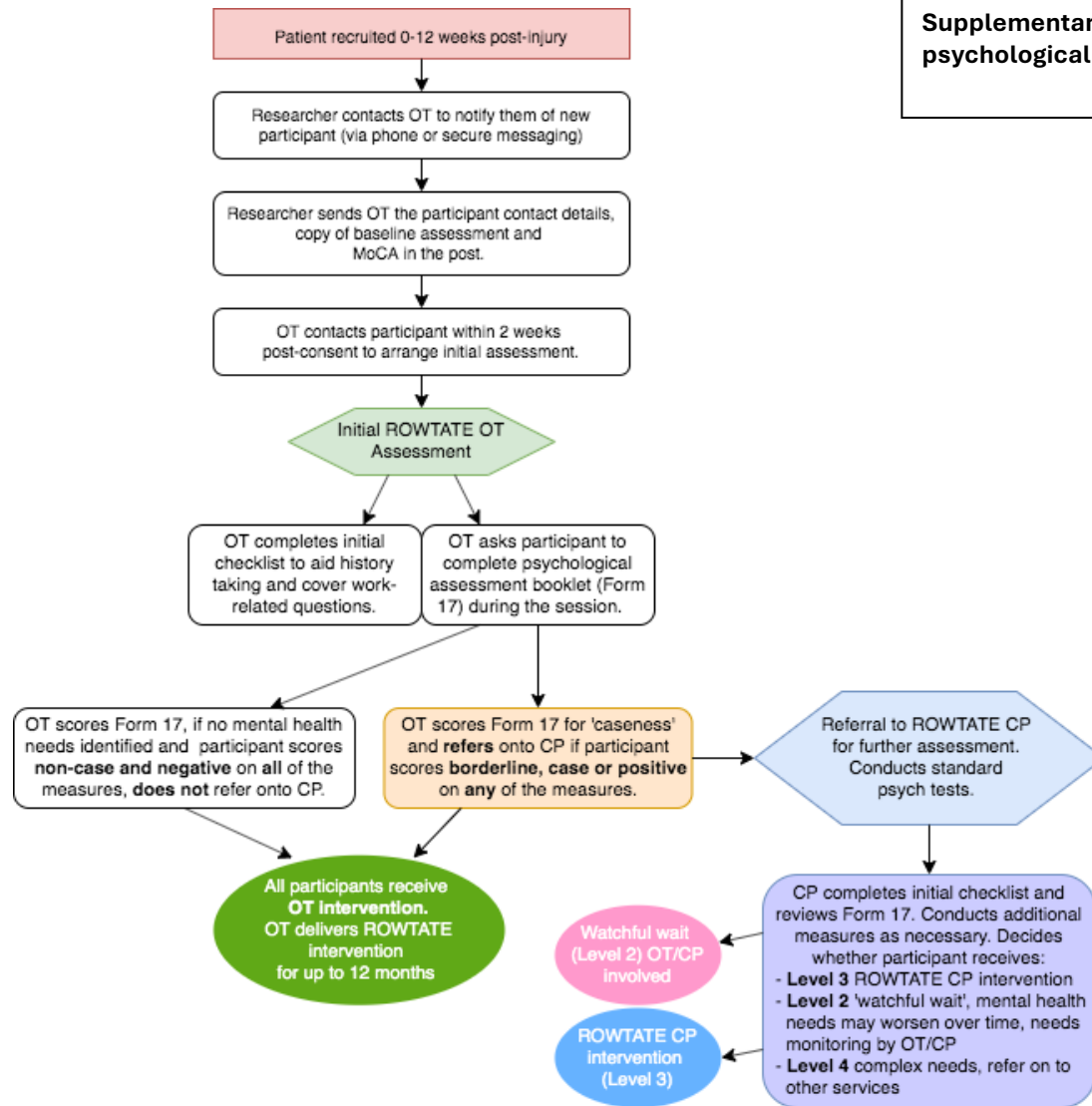

Supplement: online supplemental figure 4 [file bmjopen-14-10-s005.pdf]
