# Supplementary material for: Development of a vocational rehabilitation intervention to support return-to-work and well-being following major trauma: a person-based approach
Source: BMJ Open. 2024 Oct 4;14(10):e085724. doi: 10.1136/bmjopen-2024-085724 (PMC11459317; doi:10.1136/bmjopen-2024-085724)
Supplement: Supplementary file 6 [file bmjopen-2024-085724.reviewer_comments.pdf]

PEER REVIEW HISTORY

BMJ Open publishes all reviews undertaken for accepted manuscripts. Reviewers are asked to complete a checklist review form (**http://bmjopen.bmj.com/site/about/resources/checklist.pdf**) and are provided with free text boxes to elaborate on their assessment. These free text comments are reproduced below.

ARTICLE DETAILS

**TITLE (PROVISIONAL)**	Development of a Vocational Rehabilitation Intervention to support Return to Work and Wellbeing following Major Trauma: A Person-Based Approach
**AUTHORS**	Radford, Kate; Kettlewell, Jade; das Nair, Roshan; Morriss, Richard; Holmes, Jain; Kellezi, Blerina; Timmons, Stephen; Jones, Trevor; Tresidder, Hereward; Andrews, Isobel; Bridger, Kay; Patel, Priya; Lindley, Rebecca; De Dios Perez, Blanca; Statham, Abigail; Jones, Tadeusz; Hoffman, Karen; James, Marilyn; Kendrick, Denise; On behalf of, The Rowtate Study Team

VERSION 1 – REVIEW

**REVIEWER**	Kus, Sandra<br>Ludwig-Maximilians-Universität München, Chair of Public Health and Health Services Research
**REVIEW RETURNED**	22-Apr-2024

**GENERAL COMMENTS**	I would like to express my gratitude for the opportunity to review this impressive work.<br><br>In their work, the authors describe the extremely comprehensive and well-founded iterative process of developing a VR intervention for trauma patients. The development process is based on the Medical Research Council (MRC) guidance for the development of complex interventions and was carried out in consecutive or parallel phases with differentiated objectives and outcomes. The procedure is described in the manuscript in an extremely clearly structured and comprehensible manner with appropriate depth. The development process, materials used, and the outcomes of individual project phases are presented in great detail, with the use of tables, figures, and comprehensive supplementary files.<br><br>On page 39 of the PDF file available for the review, Table 2 and Table 3 overlap, which presumably occurred during the creation of the PDF. Likewise, the ROWTATE Logical Model on page 56 of the PDF is not displayed correctly.<br><br>The authors are invited to consider the following minor comments:<br><br>Abstract:<br>- It would be beneficial to also add results regarding the training package.<br>- I would suggest including the number of IDWG members in the results section.<br><br>Introduction:

	- The relevance of a biopsychosocial perspective for RTW after trauma was demonstrated in the icfPROreha project, which included 775 trauma patients (Kus S, Oberhauser C, Simmel S, Coenen M. ICF-based prediction of return to work after trauma rehabilitation: Results of the icfPROreha study in patients with severe musculoskeletal injuries. Frontiers in Rehabilitation Sciences 2022: 3:960473; https://doi.org/10.3389/fresc.2022.960473). This could potentially enhance the presentation of the current state of research in the introduction.<br><br>Methods:<br>- Stage 2 (Page 6 in the body of manuscript, lines 34/35): The authors report that the topic guide was "informed by the International Classification of Functioning, Disability and Health (ICF)". Does this mean that contextual factors have been taken into account? This should be clarified to the reader.<br>- Stage 2, (Page 7 in the body of manuscript, lines 7/8): Mapping to the ICF: Did the authors refer to the linking rules published by Cieza et al. (2019)? The mapping could be described in more detail.<br><br>Results:<br>- I see a limitation in the lack of variance in the study population in the studies included for synthesis in the literature review. However, the author already addressed this issue in the limitations section.<br><br>I have no further comments on the discussion and conclusion.

**REVIEWER**	Jing, Zhi<br>Ningxia University
**REVIEW RETURNED**	26-May-2024

**GENERAL COMMENTS**	The study presents a meticulous examination of individuals engaged in post-trauma recovery, and the research is both significant and indispensable. However, there is room for optimizing the article's structure to enhance accessibility to a broader international readership, particularly among Asian scholars.

VERSION 1 – AUTHOR RESPONSE

Reviewer: 1<br>Comments to the Author:<br>I would like to express my gratitude for the opportunity to review this impressive work.<br><br>In their work, the authors describe the extremely comprehensive and well-founded iterative process of developing a VR intervention for trauma patients. The development process is based on the Medical Research Council (MRC) guidance for the development of complex interventions and was carried out in consecutive	We thank reviewer 1 for these kind comments.

or parallel phases with differentiated objectives and outcomes. The procedure is described in the manuscript in an extremely clearly structured and comprehensible manner with appropriate depth. The development process, materials used, and the outcomes of individual project phases are presented in great detail, with the use of tables, figures, and comprehensive supplementary files.	
On page 39 of the PDF file available for the review, Table 2 and Table 3 overlap, which presumably occurred during the creation of the PDF. Likewise, the ROWTATE Logical Model on page 56 of the PDF is not displayed correctly.	Thank you for this comment. We will endeavour to ensure the files are correctly uploaded on re-submission
Abstract:<br>- It would be beneficial to also add results regarding the training package.<br>- I would suggest including the number of IDWG members in the results section.	We have added the following text to the abstract;<br>*Stage 4:* meetings (n=7) with intervention development working group (IDWG) to the methods and the following to the results section of the abstract.<br>'Intervention features identified by IDWG members (n=13) from stages 1 and 2 were'….:
Introduction:<br>- The relevance of a biopsychosocial perspective for RTW after trauma was demonstrated in the icfPROreha project, which included 775 trauma patients (Kus S, Oberhauser C, Simmel S, Coenen M. ICF-based prediction of return to work after trauma rehabilitation: Results of the icfPROreha study in patients with severe musculoskeletal injuries. Frontiers in Rehabilitation Sciences 2022: 3:960473; https://doi.org/10.3389/fresc.2022.960473). This could potentially enhance the presentation of the current state of research in the introduction.	Thankyou for this suggestion, we have added the following to the introduction;<br>The relevance of a biopsychosocial perspective for RTW after trauma was demonstrated in the icfPROreha study. Kus et al, (ref) assessed predictors of RTW at 78 weeks post-discharge from trauma rehabilitation in a prospective multicentre longitudinal study involving 761 patients with large joint injuries and complex fractures. they identified multiple psychosocial predictors of return to work, alongside health and disability related factors, recognising the need for a biopsychosocial approach to addressing RTW and person-centered rehabilitation. Predictors included professional sector (working in the construction, architecture, surveying, and building services engineering), ongoing legal disputes, financial concerns, personality traits, pre-accident life satisfaction, attitude to life, and demand for pension claim. Health and disability predicters were general health, current state of health, sensation of pain, limitations and restrictions in activities and participation.

Methods:<br>- Stage 2 (Page 6 in the body of manuscript, lines 34/35): The authors report that the topic guide was "informed by the International Classification of Functioning, Disability and Health (ICF)". Does this mean that contextual factors have been taken into account? This should be clarified to the reader.	Thankyou – yes it took account of these contextual factors. We have calrrified this by adding;<br><br>A topic guide, informed by the International Classification of Functioning, Disability and Health (ICF)[52], thus taking account of contextual factors affecting RTW and by our previous research in stroke[33] and TBI[34], (See Supplemental File 1), ….
- Stage 2, (Page 7 in the body of manuscript, lines 7/8): Mapping to the ICF: Did the authors refer to the linking rules published by Cieza et al. (2019)? The mapping could be described in more detail.	Thank you for alerting us to this. We did not refer to the Linking rules, published by Cieza. However, we have acknowledged this as a limitation to the discussion as follows;<br><br>Finally, qualitative data collected in stage 2 could have been mapped to ICF constructs by implementing ICF linking rules recommended by Coenen et al. (2006)[99] and Cieza et al. (2019)[100]. This may have facilitated data interpretation and analysis. Future studies developing biopsychosocial interventions should consider implementing ICF linking rules to enhance communication among healthcare providers, researchers, policymakers and patients.<br><br>Further detail of the analysis and ICF mapping have been added as follows and a new supplemental file reflecting the **Qualitative data coding framework linked to the ICF contextual factors**<br><br>Interviews were recorded, transcribed by a University of Nottingham approved transcription service, and independently analysed by three researchers (JK, KB, PP). Data were thematically analysed using Nvivo™ version 12[53] adopting a staged approach, involving both inductive independent coding and theme development driven by the data to ensure trustworthiness[57] and deductive coding informed by the contextual factors (environmental and personal) of the ICF[53] to characterise biopsychosocial and contextual influences on participants return to work. The coding framework informed by the ICF is shown in Supplemental Table 3.<br><br>Codes were categorised and organised into themes and subthemes, and agreed with four

	authors (KB, JK, KR, BK) then summarised narratively, and a map of themes and subthemes created for discussion with PPI and the wider research team. Where disagreements arose, analyses were discussed until consensus was reached. The transcripts were revisited to ensure the accounts were coherent and accurately reflected the dataset. This combined approach ensured coding and theme development were data-led, allowing for any important, unexpected issues to be identified, whilst enabling the biopsychosocial and contextual issues and challenges trauma survivors face in returning to work to be described to inform intervention development. The summary of themes was to inform development of the guiding principles. Findings from interviews and focus groups informed the iterative development of the logic model, participant-based resource use measurement capture and outcome measures for use in the feasibility study and future RCT.
Results:<br>- I see a limitation in the lack of variance in the study population in the studies included for synthesis in the literature review. However, the author already addressed this issue in the limitations section.	Thank you
Reviewer: 2<br>Comments to the Author:<br>The study presents a meticulous examination of individuals engaged in post-trauma recovery, and the research is both significant and indispensable. However, there is room for optimizing the article's structure to enhance accessibility to a broader international readership, particularly among Asian scholars.	We thank reviewer 2 for this comment. We recognise the importance of optimising the article's structure to enhance accessibility for a broader international readership, especially among Asian scholars.<br><br>We have structured the article using examples from other published studies adopting a Person Based Approach to intervention development, and followed what we consider to be a logical, temporal sequence of developmental stages underpinned by the MRC Framework. We are unclear how to structure this in a way that might make it more accessible to Asian Scholars or a broader international readership.<br><br>Therefore, no structural changes have been made. However, we hope that the content of our manuscript still provides valuable insights to complex intervention development and

	contributes meaningfully to the field. Thank you once again for your valuable comments.
